# Basin dependencies of tropical cyclone genesis environment and possible future changes revealed by machine learning methods

**DOI:** 10.1016/j.isci.2024.111714

**Published:** 2025-01-03

**Authors:** QiFeng Qian, YeFeng Chen, XiaoJing Jia, Hao Ma, Wei Dong

**Affiliations:** 1 Zhejiang Institute of Meteorological Science, HangZhou, Zhejiang, China; 2 Key Laboratory of Geoscience Big Data and Deep Resource of Zhejiang Province, School of Earth Sciences, Zhejiang University, HangZhou, Zhejiang, China; 3 Zhejiang Climate Center, HangZhou, Zhejiang, China

**Keywords:** Natural sciences, Earth sciences, Applied sciences

## Abstract

Tropical cyclone (TC) genesis mechanisms remain debated, complicating predictions of climate change impacts. This study uses principal-component analysis (PCA), confidence ellipses, and correlation circles to analyze TC genesis environments across ocean basins. Results show that TC genesis is basin dependent, except in the North Atlantic (NA), where absolute vorticity primarily drives differences in genesis locations. Ocean basins are categorized into three groups based on PCA, and three MaxEnt machine learning (ML) models are developed to predict TC genesis under future scenarios. The ML models consistently project robust basin-specific TC genesis trends, demonstrating their utility in such studies. A multivariate environmental similarity analysis indicates significant climate change impacts on TC genesis environments globally, with the weakest changes in the NA. These findings underscore the critical role of absolute vorticity in TC genesis and highlight basin-specific differences in future environmental changes.

## Highlights

• The TC genesis environment is mainly influenced by absolute vorticity in most basins
• The MaxEnt models consistently project a trend in TC genesis across each basin
• Climate change significantly influences TC genesis environments in most basins

## Graphical abstract

**Figure:**
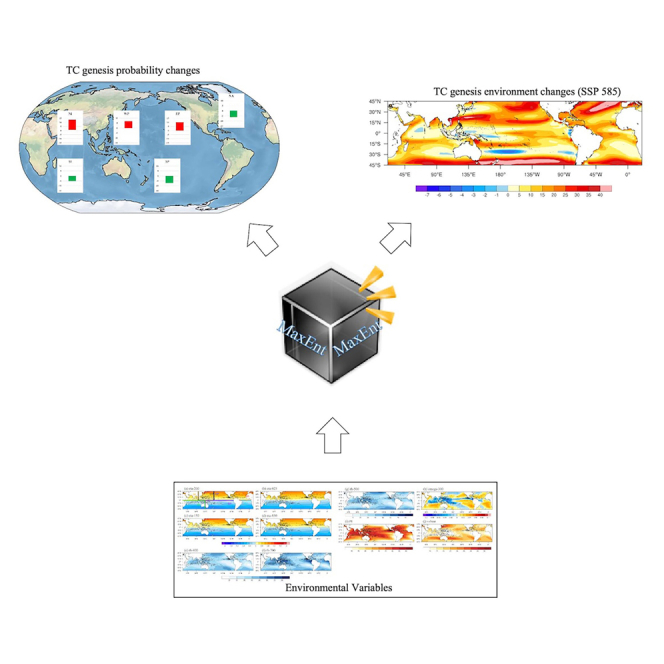

## Introduction

Tropical cyclones (TCs) are among the most devastating weather phenomena globally, impacting human life, economics, and the environment.^1^^,^^2^^,^^3^^,^^4^ Approximately 80 ± 10 TCs occur annually worldwide,^5^^,^^6^ yet no theory exists to explain this frequency.^3^^,^^4^ Gray^7^ initially demonstrated the sensitivity of TC genesis to various environmental factors. Subsequent research has identified many variables influencing TC genesis, including upper and lower atmospheric vorticity,^8^^,^^9^ atmospheric static stability,^10^^,^^11^^,^^12^^,^^13^ mid-level tropospheric humidity deficit,^14^^,^^15^ vertical wind shear,^14^^,^^16^^,^^17^ and potential intensity (PI).^18^^,^^19^ These variables are considered crucial factors affecting TC genesis.^14^ Empirical indices,^16^^,^^20^^,^^21^ TC detection algorithms,^22^ and machine learning (ML) models^23^ have been developed using these environmental variables to analyze variations in TC genesis.

In recent years, the potential changes in TC genesis due to climate change have garnered growing interest.^1^^,^^2^ While various techniques have been developed to explore these changes, the conclusions are still controversial and lack consensus. Some studies suggest an increase in the number of TCs globally,^24^^,^^25^ while others indicate either a decrease or no change.^1^^,^^2^^,^^26^ These controversial results are mainly due to the following reasons: (1) lack of consensus on TC genesis theory^4^^,^^13^ and (2) low confidence in observational long-term (multidecadal to centennial) trends of TC genesis.^2^ Nevertheless, the total number of global TCs is expected to decrease or remain unchanged with a medium confidence level.^2^

Compared to the global scale, the future projections of basin-wide TC genesis trends are even more contentious and less robust.^2^^,^^3^ The differences in TC genesis environment on an ocean basin-wide scale are poorly understood.^27^ Previous studies revealed that, while some large-scale climate variability modes may concurrently influence TC genesis variations across different basins,^4^^,^^26^^,^^27^^,^^28^^,^^29^^,^^30^^,^^31^^,^^32^^,^^33^ overall, TC genesis fluctuations in different ocean basins tend to be independent.^34^ Menkes et al.^35^ demonstrated that the performance of various TC genesis indices depends on the ocean basins and timescales considered. Bruyère et al.^36^^,^^37^ noted that TC genesis over the North Atlantic (NA) is closely related to the environment over the main development regions.^38^ However, the resemblances and differences in TC genesis environments across various ocean basins remain unclear and need further investigation. Figuring out the crucial factors that impact the TC genesis environments for different ocean basins may help to clarify the reasons for the controversial results on the TC genesis changes obtained by previous studies. It may also provide critical information for improving the prediction of ocean basin-wide TC genesis in climate models.

In recent years, ML has gained popularity in climate research and has proven helpful for understanding and predicting climate variations.^23^^,^^39^^,^^40^^,^^41^^,^^42^ Our previous work^23^ used an ML model (the maximum entropy model; MaxEnt hereafter) to predict future TC genesis probability. This MaxEnt model projected a robust decreasing trend in TC genesis probability under all shared socioeconomic pathway (SSP) scenarios. However, the projected trends for basin-wide TC genesis probability are less robust, consistent with findings in previous studies.^3^ Nevertheless, the underlying reasons for this phenomenon remain unclear.

This study employs principal-component analysis (PCA) to investigate the similarities and differences in the TC genesis environment across all ocean basins. Subsequently, we classify the basins into three groups based on the PCA results and train three MaxEnt models accordingly. Our results indicate that these MaxEnt models consistently predict changes in TC genesis probability. Finally, we utilize a multivariate environmental similarity surface (MESS) to explore the possible changes in the TC genesis environment in the Coupled Model Intercomparison Project phase 6 (CMIP6) models across the ocean basins.

## Results

### The basin-wide TC genesis environment

The dataset of ten environmental variables that are important for TC genesis, as revealed by previous work, is obtained from the National Centers for Environmental Prediction (NCEP)-National Center for Atmospheric Research (NCAR) Reanalysis I,^43^ including absolute vorticity (eta; 150 hPa, 200 hPa, 850 hPa, and 925 hPa), vertical velocity (omega; 300 hPa), relative humidity (rh; 400 hPa, 500 hPa, and 700 hPa), PI, and vertical wind shear (vshear; between 200 hPa and 850 hPa) data. PCA was applied to all environmental variables during 2000–2009. We select the first two principal components (PCs) based on their explained variances and correlation with environmental variables. Then, using the International Best Track Archive for Climate Stewardship (IBTrACS),^44^ we identify TC genesis locations across various ocean basins from 2000 to 2009, plotting them in the PC space. We analyze the period from 2000 to 2009 for two primary reasons. (1) Changes in the technology used to collect TC best-track data have introduced uncertainties in the observational TC frequency trends, resulting in low confidence.^2^ Therefore, TC tracks from more recent years are more suitable for training. (2) It is crucial to validate the performance of the trained MaxEnt models when applied to CMIP6 data. However, the CMIP6 climate models’ historical runs ended in 2014. Therefore, the period from 2000 to 2009 is the most recent time frame covered by the CMIP6 historical runs, making them essential for our research.

**Figure 1:**
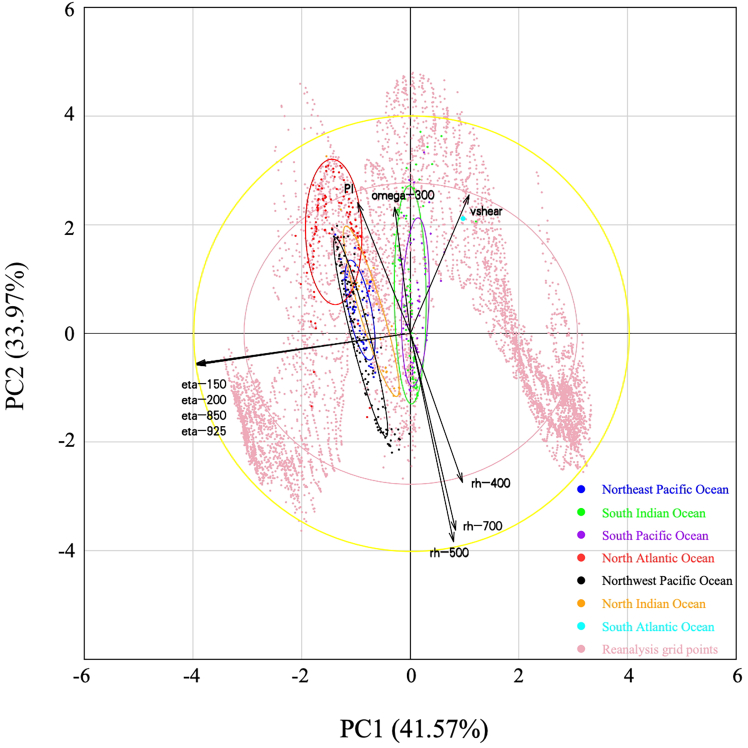
TC genesis locations of different ocean basins and reanalysis grid points (dots) and corresponding confidence ellipses (95% confidence level) during 2000–2009 in PC space The yellow circle and black vector present the results from the correlation circle, which is enlarged by a factor of four for better presentation.

Figure 1 shows TC genesis locations for all ocean basins and PC space reanalysis grid points. Additionally, we depict confidence ellipses and correlation circles for these TC genesis locations in the PC space. Firstly, confidence ellipses for the four ocean basins in the Northern Hemisphere (including the North Indian Ocean [NI], Northwest Pacific Ocean [WP], Northeast Pacific Ocean [EP], and North Atlantic Ocean [NA]) are distinct from those of the two ocean basins in the Southern Hemisphere (including the South Indian Ocean [SI] and the South Pacific Ocean [SP]). We exclude the South Atlantic Ocean basin due to only one TC generated. The confidence ellipses of the three oceans in the Northern Hemisphere, i.e., the NI, WP, and EP, largely overlap, indicating that the TC genesis environments in these ocean basins are similar. Similarly, the TC genesis environment in the SI resembles that in the SP. However, the confidence ellipse of the NA (red ellipse) is distinctly separated from the other ocean basins, with only minor overlap with the WP, EP, and NI, suggesting that the TC genesis environment over the NA may differ from that in other basins.

Furthermore, except for the NA, TC genesis locations generally align with the direction of the absolute vorticity (i.e., eta vectors). Positive absolute vorticity corresponds to the WP, EP, and NI, while negative absolute vorticity corresponds to the SI and SP, indicating that the sign of absolute vorticity primarily governs differences in TC genesis locations in the ocean basins of the two hemispheres. Contrasting with other ocean basins, the NA confidence ellipse predominantly aligns with the positive direction of the PI and vertical velocity (omega-300) and the negative direction of the relative humidity (rh). This suggests that the TC genesis environment in the NA features higher PI and mid-level vertical velocity alongside lower mid-to-low-level relative humidity compared to the WP, EP, and NI.

In summary, the above PCA analysis indicates that TC genesis locations in ocean basins are primarily influenced by absolute vorticity. However, the TC genesis environment in the NA differs from those in other ocean basins. Based on these results, we categorize the TC genesis environment into three groups: the EP, WP, and NI form one group; the SI and SP constitute another group; and the NA represents a distinct group.

### The construction and validation of three MaxEnt models

In this section, we train three MaxEnt models using ten environmental variables from the NCEP-NCAR Reanalysis dataset. Subsequently, after undergoing several validation processes, these MaxEnt models are transferred to use the CMIP6 model output. The training and validation procedures for each MaxEnt model follow the methodology of Qian et al.^23^ Specifically, considering the similarity of the TC genesis environment across the EP, WP, and NI, we bilinearly interpolated the ten variables to TC genesis locations in these regions. We then use these data to train the first MaxEnt model (EP_WP_NI) to separately capture the relationship between TC genesis probability and environmental variables in these three ocean basins. Similarly, ten variables from TC genesis locations in the SI and SP are employed to train the second MaxEnt model (denoted as SI_SP), while we use ten variables from TC genesis locations in the NA to train the third MaxEnt model (denoted as NA).

The performances of the three MaxEnt models are initially validated using the receiver operating characteristic (ROC) curve. All three MaxEnt models demonstrate an area under the ROC curve (AUC) value exceeding 0.95, significantly surpassing random prediction (AUC = 0.5) and approximately reaching 1.00. This result suggests that the three MaxEnt models are reliable and can be used to predict the TC genesis probability (Figure S7). In addition, dividing the NCEP-NCAR Reanalysis dataset into three 10-year periods (1990–1999, 2000–2009, and 2010–2019), the three MaxEnt models demonstrate comparable predictive abilities in estimating the basin-wide observational TC genesis distribution across all three subperiods, as indicated by comparison with two genesis potential index (GPI) models (Table S2). This finding further reinforces the proficiency of the three MaxEnt models in predicting TC genesis probability. Following the aforementioned validation, we then provide the corresponding environmental variables from the historical runs of five CMIP6 models for the period 2000 to 2009 to the three MaxEnt models. These five CMIP6 models are selected as they cover all the SSP scenarios.^23^ The predicted basin-wide TC genesis probability exhibits high spatial correlation coefficients with observed TC numbers during this period, indicating that the three MaxEnt models can effectively replicate observational TC genesis patterns using CMIP6 model output.

### The predicted changes in the basin-wide TC genesis probability

**Figure 2:**
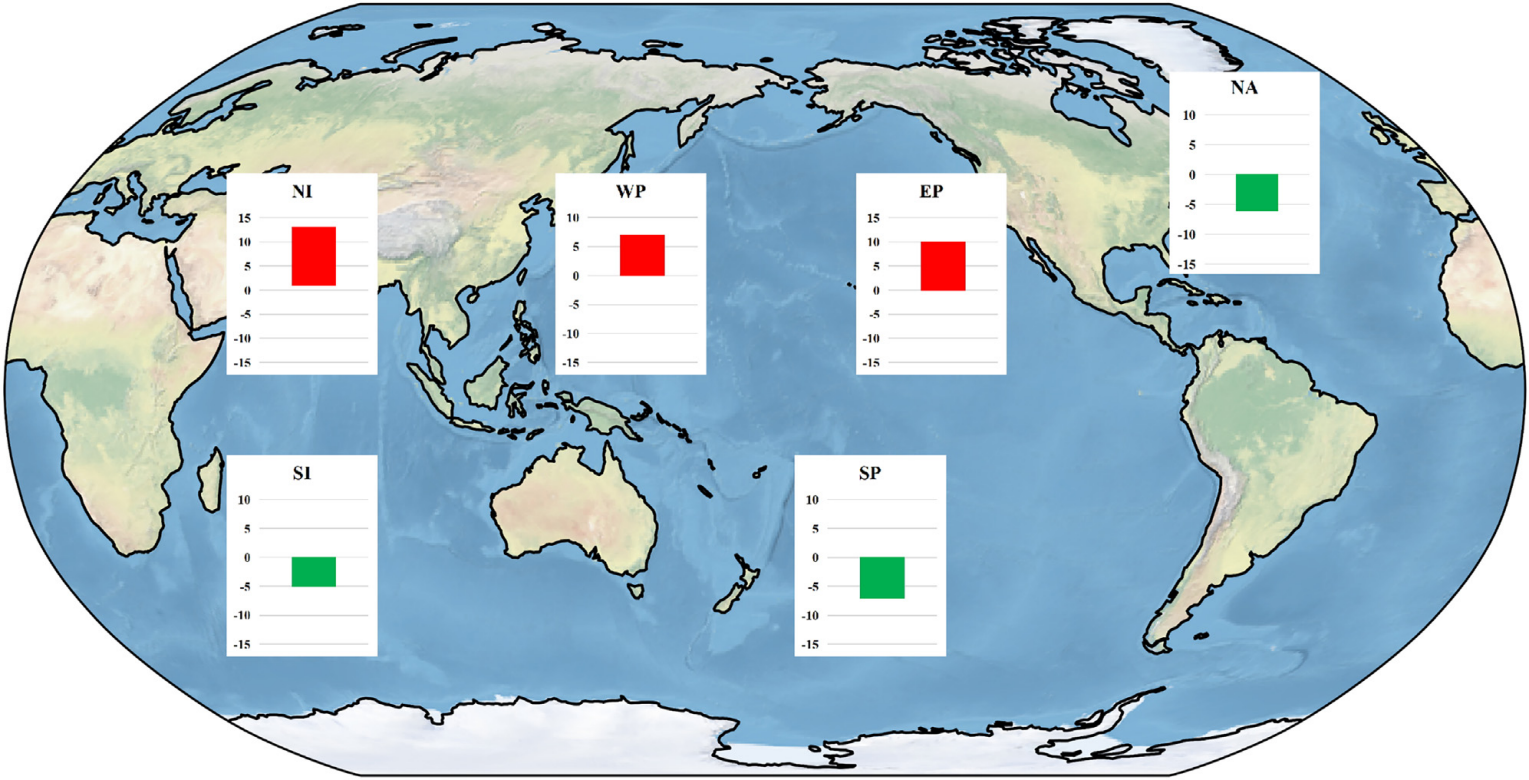
MaxEnt model predictions of TC genesis probability changes between the 2100s and the 1850s under all SSP scenarios using five CMIP6 models The bar represents the data range, with the lower edge of the bar representing the minimum and the upper edge representing the maximum.

After completing the aforementioned validations, the environmental variables obtained from CMIP6 model outputs, with a 10-year interval from 1860 to 2100, are utilized for the three MaxEnt models to predict TC genesis probability. Figure 2 illustrates the range of TC genesis probability changes in the 2100s under all SSP scenarios compared to the 1850s. The three MaxEnt models consistently predict the same trend signs under different SSP scenarios using various climate models. Specifically, the MaxEnt models forecast TC genesis probability changes ranging from 1% to 12% in the NI, 0%–7% in the WP, 0%–10% in the EP, −6%–0% in the NA, −5%–0% in the SI, and −7%–0% in the SP for the 2100s. Similar to the findings by Qian et al.,^23^ the severity of climate warming correlates with an increase or decrease in TCs generated in individual basins. Previous studies comparing model projection results across all ocean basins have noted that only the decrease in the SP and SI is relatively robust.^2^^,^^3^ Our results are consistent with this previous research; however, our MaxEnt models demonstrate robust predictions across all ocean basins.

The relative contributions of environmental variables to the three MaxEnt models in predicting TC genesis probability are examined. The percentages of the contributions of the ten variables to the three MaxEnt models are presented in Table 1. It shows that PI has the largest contribution to the three MaxEnt models. Unlike Qian et al.,^23^ which shows that PI has a much larger contribution than all the other nine variables, low-level absolute vorticity (eta-850, eta-925) also has a large contribution to EP_WP_NI, SI_SP, and NA. Overall, the results emphasize the importance of environmental variables of PI and low-level absolute vorticity in MaxEnt model predictions. Moreover, the response curve analysis further confirms the significance of PI as one of the most influential environmental variables for the EP and SP basins. In other ocean basins, the MaxEnt model predictions are primarily influenced by absolute vorticity, with other variables also contributing to some extent. Our MaxEnt model results further highlight that the TC genesis environment is basin dependent.

**Table 1:** The contribution of each environmental variable to each MaxEnt model (unit: %)

	EP_WP_NI	SI_SP	NA
PI	41.80	37.80	37.00
vshear	2.20	5.80	8.60
omega-300	7.90	2.20	2.10
rh-400	0.30	1.40	5.00
rh-500	0.70	1.90	14.80
rh-700	2.20	6.60	1.20
eta-150	8.20	5.80	0.40
eta-200	5.20	0.20	0.60
eta-850	1.60	37.70	0.70
eta-925	29.90	0.50	29.70

The values are calculated for the trained model for the period 2000–2009.

### The predicted changes in the basin-wide TC genesis environment

In this section, we apply MESS analysis to the five CMIP6 model outputs to evaluate the differences in TC genesis environments between two reference time periods. The reference period for MESS analysis is the 1850s, while the target period is the 2100s. The differences imply the impact of climate changes on the TC genesis environments between these two periods. A positive MESS value indicates that the CMIP6 model-simulated TC genesis environment in the 2100s is similar to that in the 1850s (with larger values indicating more significant similarity). In contrast, a negative MESS value suggests that the TC genesis environment simulated by CMIP6 models in the 2100s differs from that in the 1850s, indicating changes due to global climate change.

**Figure 3:**
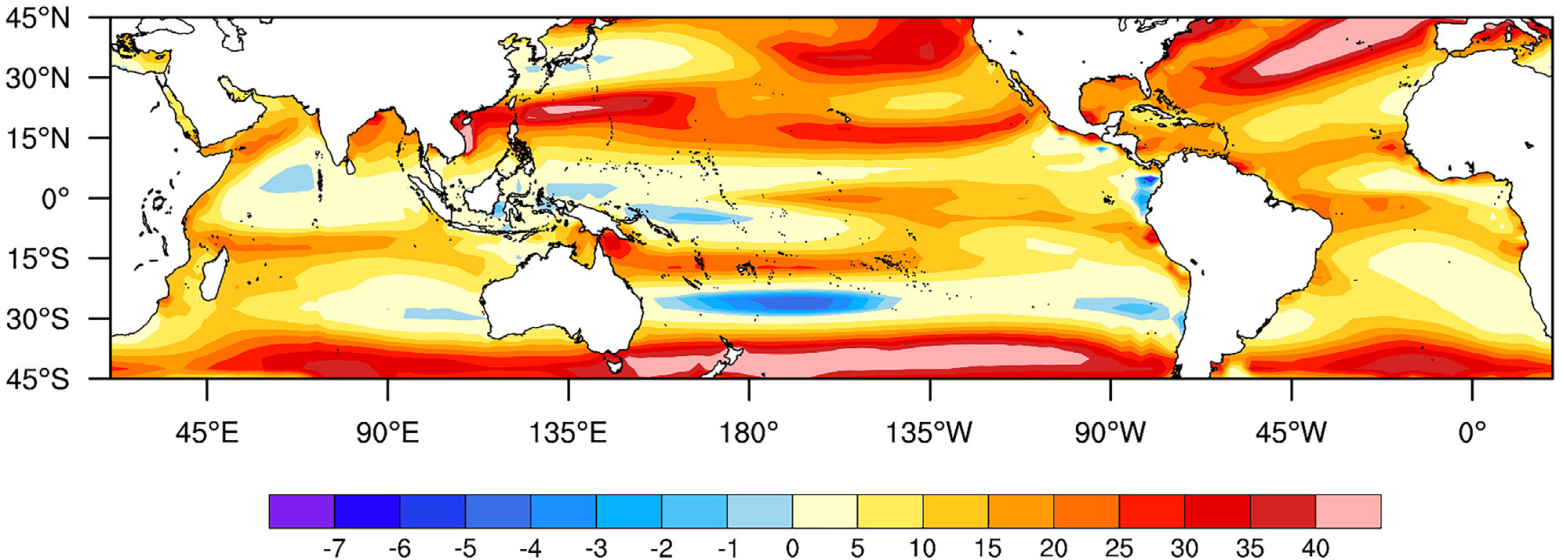
The ensemble means of the MESS analysis of five CMIP6 models under the SSP 585 scenario

Figure 3 illustrates the ensemble means of the MESS values for the five CMIP6 model projections under the SSP585 scenario. The SSP585 scenario represents a socioeconomic pathway in which the global economy is heavily reliant on fossil fuels for energy, resulting in the most severe climate change experienced by the Earth. Negative MESS values are evident in all ocean basins except the NA under this scenario. In contrast, large positive MESS values dominate extensive regions in the NA, indicating that the simulated TC genesis environment in the 2100s resembles that of the 1850s. This suggests that the impact of climate change on the TC genesis environment is comparatively weaker in the NA than in other ocean basins. In other words, the TC genesis environment in the NA remains relatively stable across all SSP scenarios.

We present the results for other SSP scenarios in Figure S13. In the mildest case (SSP119 scenario, which represents that the world experiences the mildest climate change), negative MESS values are only observed in the WP and SP basins in the 2100s (Figure S13A). This suggests that the predicted TC genesis environment in the WP and SP basins tends to differ from that in the 1850s, indicating a significant impact of climate change under the SSP119 scenario on TC genesis in these two basins. In contrast, under this scenario, other basins will experience a similar TC genesis environment in the future. It also noted that negative MESS values begin to emerge worldwide with the increasing severity of climate change.

## Discussion

Previous studies have revealed that the mechanisms of basin-wide TC genesis are more complex and less understood compared to global-scale TC genesis. Therefore, projections of changes in basin-wide TC genesis under the influence of global climate change entail more significant uncertainties. ML techniques, capable of capturing complex nonlinear relationships, have proven valuable tools for elucidating information to understand the nature of TC genesis in various ocean basins, particularly without a well-established TC genesis theory.

In this study, we first apply PCA, confidence ellipses, and correlation circle to examine the TC genesis environment across different ocean basins. Our results indicate that, aside from the NA, the TC genesis environments in both the Northern Hemisphere and Southern Hemisphere are primarily influenced by the sign of absolute vorticity. Conversely, the TC genesis environment in the NA is characterized by higher PI and mid-level vertical velocity, along with lower low- and mid-level relative humidity compared to other ocean basins in the Northern Hemisphere. Prior research has also highlighted the unique features of TC genesis in the NA. For instance, Bruyère et al.^37^ observed that African easterly waves served as the predominant source of seedlings for NA TCs, a pattern not observed in other regions. Additionally, Emanuel^25^ found that NA TC activity over the past century was closely linked to regional rather than global climate change, emphasizing the distinctive nature of the NA TC genesis environment. Our PCA analysis in this study further corroborates that TC genesis environments exhibit basin-dependent characteristics, with the NA displaying distinct features compared to other basins.

Subsequently, we categorized the TC genesis environment into three groups based on the PCA results and then trained three MaxEnt models using selected environmental variables from these three groups for simulation and prediction purposes. The three MaxEnt models consistently project a robust TC genesis trend with consistent signs in each basin across different SSP scenarios, underscoring the utility and reliability of these ML models for basin-wide TC genesis investigation and predictions. Further analysis identified PI and low-level absolute vorticity as crucial variables for predicting the three MaxEnt models. Previous studies have underscored the significant role of low-level vorticity in TC genesis. Tippett et al.^20^ developed a Poisson regression index for TC genesis, demonstrating its response to low-level absolute vorticity saturating when exceeding a threshold. Ge et al.^45^ conducted cloud-resolving numerical simulations and revealed that, given the same column integrated absolute vorticity, a lower-level vortex has a much higher TC generation efficiency than an upper-level vortex. Hsieh et al.^46^ further emphasized the essential role of convection processes for TC genesis under a low-frequency, low-level vorticity background. Moreover, Hsieh et al.^47^ pointed out that low-level vorticity’s high-frequency component primarily influences TC genesis’s timing and location in the WP. Additionally, Ikehata and Satoh^48^ highlighted the correlation between Northern Hemisphere TC seed frequency and low-level vorticity and vertical velocity, further emphasizing the importance of low-level vorticity in basin-wide TC genesis. These results highlight the importance of lower-level vorticity and support the findings of this study. In contrast, upper-level vorticity contributes relatively less to the three MaxEnt models than low-level vorticity. Wu et al.^8^ highlighted the influence of the tropical upper tropospheric trough (TUTT) in the North Pacific Ocean on TC genesis, with Wang and Wu^9^ further identifying TUTT through a vorticity maximum in the upper atmosphere. Recently, Hsieh et al.^49^ revealed that changes in TC seed frequency over the Northwest Pacific Ocean (WP) depend on projected alterations in large-scale atmospheric convection on background absolute vorticity, suggesting the indirect impact of upper-level vorticity on basin-wide TC genesis. In the current work, we further confirm that TC genesis environments across basins are primarily influenced by absolute vorticity, except in the NA, where the TC genesis environment is relatively complex.

Finally, we conducted an MESS analysis to assess potential changes in the TC genesis environment using data from five CMIP6 models. The results indicate that the TC genesis environment in the NA experiences the least pronounced changes across all SSP scenarios. This suggests that global climate change significantly impacts TC genesis environments in ocean basins, except for the NA, which experiences minimal changes.

Knutson et al.^3^ summarized numerous TC frequency projections and further pointed out that the median of most projections in all the basins decreased. The results of this study are consistent with those of the SI, SP, and NA but different in the NI, EP, and WP. In addition, our results suggest that NA may have a unique TC genesis environment, which is also consistent with Emanual.^25^ The TC genesis environment in the NA might experience the least pronounced changes under the influence of climate change. Furthermore, the contribution of each environmental variable to the three MaxEnt models implies that, for basin-wide TC genesis projection, low-level vorticity becomes essential, consistent with previous theoretical studies.^45^^,^^46^^,^^47^^,^^48^ In sum, our study provides insights into the controversial results of global-scale TC genesis changes reported in previous studies. It offers valuable information to enhance the prediction capabilities of climate models. The modeling method in this study also provides the potential to apply ML methods in investigating TC genesis-related investigations. For example, the modeling process may help to explore the TC genesis poleward migration.

Finally, the modeling approach utilized in this study demonstrates the potential of applying ML methods to investigate aspects related to TC genesis. For instance, this modeling process may facilitate exploring the poleward migration of TCs, which is crucial for understanding how TCs may respond to climate change. Furthermore, our results can inform the current understanding of TC intensification and track prediction by highlighting the environmental variables significantly influencing TC genesis. This insight is particularly relevant in the context of climate change, as shifts in these variables may alter TC behavior, intensification patterns, and associated risks. Future research could enhance predictive capabilities concerning TC activity and its implications for coastal communities and ecosystems by integrating ML techniques with traditional climate analysis.

### Limitations of the study

In this study, we emphasize the robustness of our ML model predictions in investigating the environments associated with TC genesis. However, inherent uncertainties and limitations exist. For example, the sensitivity of the MaxEnt models to biases in TC genesis locations remains to be determined and requires further investigation. In our work, the MaxEnt models are based on ten-year climatological averages of environmental variables. Future research could explore whether these models can effectively assess the seasonal cycles of TC genesis and provide deeper insights into this phenomenon. It is important to note that MaxEnt is an ML model that does not capture the interplay among environmental variables. Therefore, employing advanced techniques such as few-shot learning may provide a more comprehensive understanding of these interactions.

## Resource availability

### Lead contact

Further information and requests for resources should be directed to the lead contact, XiaoJing Jia (jiaxiaojing@zju.edu.cn).

### Materials availability

This study used public datasets and did not generate new datasets.

### Data and code availability

• This study analyzes public datasets. All the datasets used in this study can be obtained from corresponding official website. Links are listed in the key resources table.
• The software and code used in this study are open-source packages, and the corresponding links are also listed in the key resources table.
• Any additional information needed is available from the lead contact upon request.

## Acknowledgments

This research was funded by the 10.13039/501100001809National Natural Science Foundation of China (grant no. 42205020), the 10.13039/501100004731Zhejiang Provincial Natural Science Foundation (grant no. LQ23D050003), and the Joint Funds of the 10.13039/501100004731Zhejiang Provincial Natural Science Foundation (grant no. LZJMD24D050002).

## Author contributions

Q.Q. conceived the original idea, developed the model, performed the study, and wrote the original draft. X.J. collected resources, supervised the study, and contributed to the writing and reviewing with important contributions from Y.C., H.M., and W.D.

## Declaration of interests

The authors declare no competing interests.

## STAR★Methods

### Key resources table

**Table:** 

REAGENT or RESOURCE	SOURCE	IDENTIFIER
**Deposited data**		

IBTrACS TC track dataset	National Centers for Environmental Information	https://www.ncdc.noaa.gov/ibtracs/
NCEP-NCAR Reanalysis I dataset	National Oceanic and Atmospheric Administration	https://psl.noaa.gov/data/gridded/
Met Office Hadley Center Sea Surface Temperature dataset	Met Office Hadley Center	https://www.metoffice.gov.uk/hadobs/index.html
CMIP6 model output dataset	Earth System Grid Federation	https://aims2.llnl.gov/search/

**Software and algorithms**		

MaxEnt and related code (open source)	Phillips et al.^50^	https://biomodhub.github.io/biomod2/
PCA and related code (open source)	Dray and Dufour^51^	https://adeverse.github.io/ade4/
MESS and related code (open source)	Elith et al.^52^	https://github.com/rspatial/dismo

### Experimental model and study participant details

Only numerical experiments are conducted in this study. No animals, human participants, plants, microbe strains,cell lines, primary cell cultures are used in this study.

The MaxEnt model used in this study is a classic machine learning model proposed following the “maximum entropy principal”.^50^ This principal reveal that the best approximation of an unknown probability distribution should be 1. Satisfy all the known constraints on the unknown distribution; 2. Have maximum entropy. In our previous work,^23^ we applied a MaxEnt model to predict the changes in global TC genesis probability under different climate scenarios, which is used to investigate the variation in TC genesis probability in basins in this study. The MaxEnt model is trained by climatological mean NCEP reanalysis data on TC genesis locations during 2000–2009 and then validated using several different methods on the NCEP dataset at different time periods. After the MaxEnt model is validated on the NCEP dataset, we deploy the environmental variables from CMIP6 model historical runs during 2000–2009 and ensure that the model output is reliable. Finally, the run outputs of the historical CMIP6 model (1850–2014), SSP 119, SSP 126, SSP 245, SSP 370, SSP 434, SSP 460 and SSP 585 (2015–2100) are supplied to the MaxEnt model to predict TC genesis in each basin in the future. In this study, we first analyze the resemblances/differences of TC genesis environment over basins and then train different MaxEnt model for basins have similar TC genesis environment to make the basin-wide TC genesis probability trend projections more consistent.

### Method details

#### Data preprocess

The data preprocess in this study are the same as those used by Qian et al.^23^ For convenience, we briefly describe them as follows.

(1) TC track data with a temporal resolution of 6 h are obtained from the International Best track Archive for Climate Stewardship (IBTrACS).^44^ The TC genesis locations are derived from the observational IBTrACS dataset for the period of 2000–2009. Following the methods of Li et al.,^53^ only storms that reach a tropical storm intensity (with maximum sustained wind speeds >34 knots) are defined as TCs. Once a TC is selected, the first location of its track is defined as its genesis location. In the current work, a total of 828 TCs are defined from 2000 to 2009 and are used in the analysis;
(2) Monthly mean sea level pressure, air temperature, vertical velocity, relative humidity, and zonal and meridional wind speed data from 2000 to 2009 with a spatial resolution of 2.5 ° × 2.5 ° are provided by NCEP-NCAR Reanalysis I^43^. Monthly mean sea surface temperature (SST) data from 2000 to 2009 with a spatial resolution of 1 ° × 1 ° are obtained from the Met Office Hadley Center.^54^ Per Qian et al.,^23^ ten environmental variables during 2000–2009 are used to perform PCA. The ten variables are the potential intensity (PI); the vertical wind shear between 850 hPa and 200 hPa (vshear); the absolute vorticity at 150 hPa (eta-150), 200 hPa (eta-200), 850 hPa (eta-850) and 925 hPa (eta-925); the vertical velocity at 300 hPa (omega-300); and the relative humidity at 400 hPa (rh-400), 500 hPa (rh-500) and 700 hPa (rh-700). The vshear is defined as $\sqrt{{\left(u850-u200\right)}^{2}+{\left(v850-v200\right)}^{2}}$. The ten variables, overlaid with the TC genesis locations for the period of 2000–2009, are presented in Figure S1;
(3) The monthly mean historical runs (1850–2014) and the shared socioeconomic pathway (SSP) 119, SSP 126, SSP 245, SSP 370, SSP 434, SSP 460 and SSP 585 runs (2015–2100) by five Coupled Model Intercomparison Project Phase six (CMIP6) models (Table S1) are used in this study. These models are selected because they provide all the abovementioned SSP scenarios at the beginning of this study, and using as many scenarios as possible can make the results more robust. Among these SSP scenarios, SSP 119 and SSP 126 are projected to lead to temperature increases of ≤2°C, while SSP 245 and SSP 434 are expected to result in temperature changes of 2°C–3°C. SSP 460, 370, and 585 are associated with temperature increases exceeding 3°C.^55^ The SSP 119 scenario indicates a growing movement toward a sustainable economy, leading to the mildest climate change. In contrast, the SSP 585 scenario reflects a socioeconomic pathway where the global economy heavily relies on fossil fuels for energy, resulting in the most severe climate change. The ten environmental variables in future scenarios are derived from five CMIP6 models. The climatological average for each model run from 1860 to 2100 is calculated with a 10-year interval (e.g., 1850–1859, …, 2090–2099);

All the data except the TC track data are interpolated onto the same 2.5 ° × 2.5 ° longitudinal-latitudinal grids as NCEP-NCAR Reanalysis I. The definition of each basin (South Indian Ocean, SI; North Indian Ocean, NI; Northwest Pacific Ocean, WP; Northeast Pacific Ocean, EP; South Pacific Ocean, SP; North Atlantic Ocean, NA; South Atlantic Ocean, SA) matches the definitions provided by the IBTrACS dataset,^44^ which can also be found in Figure S1. In this study, the area mean of TC genesis probability is calculated over each ocean basin, as shown in Figure S1.

#### Principal component analysis (PCA)

PCA is one of the most widely used multivariate statistical tools in climate research^56^^,^^57^^,^^58^ and is also known as an unsupervised machine learning method in computer science.^59^ It is widely used to perform climate data analysis, per the methods of Lorenz,^60^ and is called “empirical orthogonal function (EOF) analysis”. Typically, PCA can be applied to a single field or multiple fields in climate research.^57^^,^^58^ Applying PCA to a single climate field is usually done to determine one or more uncorrelated modes of variability while applying PCA to multiple fields is generally done to uncover meaningful joint relationships among these fields.^57^^,^^58^ In other research areas, PCA is used differently. For example, PCA can be used to identify the differences between samples in ecological and medical studies.^61^ In these applications, the PCA calculation processes are the same, while their subsequent analysis processes are quite different from those of climate applications.

Following the methods of our previous work,^23^ we assume n tropical cyclone (TC) genesis locations during a certain time period and that each TC genesis location has p environmental variables. The goal of this study is to determine the resemblances/differences between the TC genesis environments of different basins by analyzing the p environmental variables at the n TC genesis locations and then training MaxEnt models for basins that have similar TC genesis environments. The aim of this study is quite similar to some ecological studies that use PCA to identify the resemblances/differences between specific living environments (which are called ecological niches in ecology) of some species by analyzing the corresponding environmental variables at locations where individuals of these species are observed. A conceptual diagram is shown in Figure S2. In this study, we regard the TCs that are generated over a specific basin as a “species” and treat the TC genesis locations in a specific basin as “observed individuals” of this “species”. This treatment is reasonable because the variations in TC numbers in basins tend to be independent of each other.^4^^,^^34^ The specific environmental conditions of a certain basin, within which TCs can be generated over the basin, are treated as “ecological niches”. Therefore, using this treatment, we can use PCA to analyze the resemblances/differences in the TC genesis environment among different ocean basins following the processes shown in numerous ecological studies.^61^

In this study, we conduct PCA following the duality diagram.^51^ Since PCA is a mature method, there are numerous papers^51^^,^^62^^,^^63^^,^^64^ and textbooks^61^^,^^65^ describe the theory, calculation processes and plotting procedure. An overview of the PCA workflow in this study is presented in Figure S3. We first perform PCA on the observational environmental variable dataset. The PCA will reduce the spatial dimensions of the data by selecting new dimensions (principal component; PC hereafter). By analyzing the explained variance of each PC and calculating the correlations between the PC and the environmental variables, we can select the essential PCs. We then use the selected PCs to form a new PC space and plot the TC genesis locations over different ocean basins in this space. The corresponding confidence ellipses of the TC genesis locations and the correlation circles of each basin can be calculated and plotted in the PC space. The confidence ellipses and correlation circles can tell us the resemblances/differences in the TC genesis environment of different ocean basins. Information on confidence ellipses and correlation circles can be found in many textbooks^61^^,^^66^ and therefore they are only briefly introduced in the following sections.

#### Selection of remaining PCs, confidence ellipse and correlation circle

The PCA will reduce the variable space dimensions to several PCs. It is crucial to determine the remaining PCs, which represent the main features of the original variable space. The scree plot of each PC is shown in Figure S4. The first two PCs explain most of the total variances, and the variance explained by the PCs decreases significantly after PC3. The first two PCs explain up to 75.54% of the total variance, and the remaining PCs explain 24.46% of the variance.

To clarify the relationship between these PCs and various environmental variables, their correlation coefficients are presented in Figure S5A. PC1 has large correlation coefficients with absolute vorticity at the upper and lower levels of the atmosphere (eta-150, eta-200, eta-850 and eta-925). This result indicates that PC1 mainly captures the features of the absolute vorticity at the upper and lower levels of the atmosphere. PC2 has a large correlation coefficient with low- and middle-level relative humidity (rh-500 and rh-700) and has a considerable correlation coefficient with the remaining variables. This result suggests that PC2 mainly depicts the features of low and mid-level relative humidity while also capturing part of the features of the other four environmental variables. In addition, this result also suggests that these six environmental variables may be correlated. PC3 has a large correlation coefficient with middle-level vertical velocity (omega-300) and PI, which is comparable to the correlation coefficients of PC2. This result implies that the features of middle-level vertical velocity (omega-300) and PI are captured by both PC2 and PC3. Similarly, PC4 has a large correlation coefficient with middle-level relative humidity (rh-400) and vertical wind shear (vshear), which is comparable to the correlation coefficients of PC2. This result suggests that the features of middle-level relative humidity (rh-400) and vshear are captured by both PC2 and PC4. The remaining PCs are barely correlated with the environmental variables and explain a very small amount of the variance, suggesting that they are redundant and contain little information about the environmental variables. We also calculate the relative contributions of each environmental variable to the four PCs (Figure S5B), and the results are generally consistent with the correlation heatmap, which supports our analysis.

From the above results, PC1 and PC2 tend to be sufficient to be selected and used in subsequent analysis because PC1 and PC2 account for most of the total variance and can capture the main features of all the environmental variables. However, other PCs may contain useful information. Therefore, PC1, PC2, PC3 and PC4 are all used to form the PC space.

Two PCs can form a PC space. The TC genesis locations over each basin and the grid points of the reanalysis can be plotted in this PC space. The confidence ellipse can also be determined based on these locations or grid points. The basic mathematics of the confidence ellipse are briefly introduced as follows^67^:

Let $\bar{Z}$ and S be the mean and the unbiased estimate of the covariance matrix, respectively, of a random sample of size n from a bivariate normal distribution with mean μ and covariance matrix Σ. The variable $\bar{Z}-\mu$ is distributed as a bivariate normal variate with a mean of zero and a covariance of $n^{-1}\Sigma$, and it is independent of S. The confidence ellipse for μ is based on Hotelling’s $T^{2}$ statistic:

(Equation 1) $$T^{2}=n{\left(\bar{Z}-\mu \right)}^{\prime S^{-1}}\left(\bar{Z}-\mu \right)$$

A 100(1−α)% confidence ellipse for μ is defined by the equation:

(Equation 2) $${\left(\bar{Z}-\mu \right)}^{\prime }S^{-1}\left(\bar{Z}-\mu \right)=\frac{2\left(n-1\right)}{n\left(n-2\right)}F_{2,n-2}\left(1-\alpha \right)$$

where $F_{2,n-2}\left(1-\alpha \right)$ is the (1−α) critical value of an F variate with 2 and n−2 degrees of freedom. When μ is unknown, Z is considered a bivariate random variable for an observation. The variable $Z-\bar{Z}$ is distributed as a bivariate normal variate with a mean of 0 and a covariance of $\left(1+n^{-1}\right)\Sigma$, and it is independent of S. A 100(1−α)% confidence ellipse is then given by the equation:

(Equation 3) $${\left(Z-\bar{Z}\right)}^{\prime }S^{-1}\left(Z-\bar{Z}\right)=\frac{2\left(n+1\right)\left(n-1\right)}{n\left(n-2\right)}F_{2,n-2}\left(1-\alpha \right)$$

In this case, the confidence ellipse approximates a region containing a specified percentage of the population. The confidence ellipse is centered on the means, and the variance determines its width and height. The covariance sets the slope of the main axis of the ellipse.

In this study, since PCA is applied to the gridded reanalysis data, the confidence ellipse of the grided reanalysis data is centered at the original point, representing the global ocean environment the two PCs define. The confidence ellipse of the TC genesis locations of a specific ocean basin is smaller than that of the gridded reanalysis data, which denotes the particular TC genesis environment over the specific ocean basin. Note that only one TC is generated over the South Atlantic Ocean; thus, plotting the corresponding confidence ellipse is meaningless. Therefore, we depict the TC genesis environment of each basin in PC space; however, we still cannot analyze their resemblances/differences. To further investigate the TC genesis environment, we plot the correlation circles. The basics of correlation circles are introduced as follows.^68^

The correlation between a PC and an environmental variable can estimate the amount of similar information they have. Such a correlation is called a loading in the framework of PCA. The environmental variables can be plotted as points in the PC space using their loadings as coordinates. In addition, the sum of the squared loadings between an environmental variable and all the PCs equals one. Therefore, the squared loadings are more straightforward to interpret than the loadings because the squared loadings provide the proportion of the variance of the variables that the PCs explain. When the two PCs perfectly represent all the environmental variable data, the sum of the squared loadings is equal to 1, and thus, the loadings will be positioned on a circle called a correlation circle. When more than two PCs are needed to represent the data perfectly, the environmental variables will be inside the correlation circle. In other words, the closer an environmental variable is to the circle, the better it can be reconstructed by the two PCs and the more critical it is to interpret these PCs; when an environmental variable is closer to the center point of the circle, it means that it is less crucial for the two PCs. We can summarize the correlation relationship between environmental variables by plotting vectors away from the origin to environmental variable points in PC space. Specifically, when the angle between two vectors is small, the two environmental variables are positively correlated; an angle of 90° indicates that the environmental variables are not correlated, and an angle close to 180° suggests that the environmental variables are negatively correlated. Meanwhile, when a vector is close to the x- or y axes, this environmental variable is associated with the corresponding PCs.

Using the confidence ellipse and the correlation circle together, we can identify the resemblances/differences of the TC genesis environment. All resemblances/differences of the TC genesis environment in other PC spaces (Figure S6) are also presented in the PC1 and PC2 space (Figure 2 in the main text). Thus, PC1 and PC2 are selected as the remaining PCs. The main text only presents the PC1-PC2 space results and analysis.

#### Training and validation of the MaxEnt models

As shown in Figure 1 in the main text, the confidence ellipses of the Northeast Pacific (EP), Northwest Pacific (WP) and North Indian (NI) Ocean are highly overlaid with each other, indicating that the TC genesis environments in these basins are similar. Similarly, the TC genesis environment of the South Indian (SI) Ocean is similar to that in the South Pacific (SP). The confidence ellipse of the North Atlantic (NA) Ocean is quite different from that of the other ocean basins, in which few regions are overlaid with the WP, EP, and NI, suggesting that the TC genesis environment over the NA may be different from that in other basins. Therefore, the TC genesis environment of EP, WP, and NI can be classified as one group, SI and SP as one group, and NA as one group. In this section, three MaxEnt models are built according to the three groups.

#### Training process

The training process follows Qian et al.^23^ for each MaxEnt model. Finding the solution of a MaxEnt model is equivalent to minimizing the negative log likelihood problem for a Gibbs distribution23. The training process of the MaxEnt model is terminated when the change in log likelihood between iterations becomes smaller than a predefined threshold (10^−5^). There are no model selection processes in this study. The three MaxEnt models are trained with the ten environment variables from the NCEP reanalysis dataset.^23^ Since the TC genesis environment over EP, WP and NI are similar, the ten variables are bilinearly interpolated onto the TC genesis locations over EP, WP and NI and then are used to train the first MaxEnt model (denoted as EP_WP_NI). Similarly, ten variables of TC genesis locations over SI and SP are used to train the second MaxEnt model (denoted as SI_SP). The ten variables of TC genesis locations over NA are used to train the third MaxEnt model (denoted as NA). During training, we track the environmental variable that contributes most to the model using the gain defined in Qian et al.^23^ During each step of the training process, the gain of the MaxEnt model increases by changing the coefficient of a single environmental variable, and we assign the increase in the gain to this environmental variable. After the MaxEnt model is trained, each environmental variable has its value, and then we convert them to percentages.^50^ If one environmental variable has a zero value at the end of the training process, then using this variable will not increase the model gain; thus, this variable has no contribution to the model.

#### Validation process

Traditional machine learning metrics (e.g., Accuracy, Precision and Recall) may not be suitable for modeling problems in this study. This is because TC genesis location data are presence only data, which means we have only positve instances and lack negative instances. Therefore, we generated random samples and train the model to distinguish TC samples from random samples. Under this circumstances, Accuracy metric can be misleading. A model that predicts all generated random samples correctly but performs poorly on TC genesis samples can still achieve high score. Recall and Precision focus on classification performance and do not account the spatial coherence of the probability prediction.^61^ Therefore, model validation should consider both machine learning modeling accuracy and the spatial coherence of predicted probability. The validation process generally follows Qian et al.^23^ After the training process, the Receiver Operating Characteristic (ROC) analysis, true skill statistic (TSS)^69^^,^^70^ and spatial correlation coefficient are used to compare and evaluate the three MaxEnt models. Details of the ROC analysis and spatial correlation coefficient verification methods can be found in the supplement material of Qian et al.^23^ The TSS is calculated as sensitivity + specificity - 1, where sensitivity is the probability that the model will correctly classify a presence and specificity is the probability that the model will correctly classify an generated sample.

Figure S7 displays the ROC curves for each MaxEnt model. The areas under the ROC curves (AUC) for EP_WP_NI, SI_SP, and NA are 0.95, 0.97, and 0.96, respectively. All these values are significantly higher than a random prediction (AUC = 0.5) and are close to 1.00, indicating that the three MaxEnt models are reliable for predicting TC genesis probability. The True Skill Statistic (TSS) values for EP_WP_NI, SI_SP, and NA are 0.86, 0.83, and 0.83, respectively. Typically, models with TSS values greater than 0.8 are considered to demonstrate good performance.^69^^,^^70^ Overall, both the AUC and TSS metrics indicate a reasonable accuracy for the three MaxEnt models.

The spatial correlation coefficients between the TC genesis probability predicted by the MaxEnt model, the Genesis Potential Index (GPI) proposed by Emanuel and Norlan (EM GPI)^16^ and the GPI proposed by Murakami and Wang (MK GPI)^71^ and the observations over each basin for the periods of 2000–2009 are calculated (Table S2). Overall, the spatial correlation coefficients between the TC genesis probability predicted by the MaxEnt model and the observation are comparable to that between two GPI and the observations over each basin, indicating that the MaxEnt model is reliable during 2000–2009.

To further examine the reliability of the MaxEnt model, the environmental variables during the other two periods, i.e., 1990–1999 and 2010–2019, are deployed to the pre-trained MaxEnt model. Again, the spatial correlation coefficients between the MaxEnt-predicted TC genesis probability and the observed TC frequency for 1990–1999 and 2010–2019 are high and comparable to that of the two GPI (Table S2). This result suggests that the performance of the MaxEnt model is not sensitive to the period of datasets fed into it and can be used to make predictions.

Before we perform prediction, we first check the performance of the MaxEnt model using the CMIP6 model output as input. We deploy the 2000–2009 environmental variables simulated by the five climate models to the MaxEnt model to generate the spatial distribution of the TC genesis probability. Then, the spatial correlation coefficients between the ensemble mean of the TC genesis probability from MaxEnt in 2000–2009 and the observed TC frequency are calculated (Table S2). Overall, the spatial correlation coefficient between the ensemble results and the observations is comparable to that using historical observation environmental variable data. This result suggests that the MaxEnt model is reliable and can be used to make predictions based on CMIP6 model output data.

The results in this section show that the MaxEnt model is robust for predicting the TC genesis probability. Then, the 10-year climatological averages of the environmental variables for the historical and SSP scenarios of CanESM5, FGOALS-g3, IPSL-CM6A-LR, MIROC6 and MRI-ESM2–0 are deployed to the MaxEnt model and make predictions.

#### Further analysis of the MaxEnt model output

To better understand and interpret the MaxEnt model, we perform two analyses. First, we apply the Jackknife test to the MaxEnt model and environment variables from 2000 to 2009 and compare the relative importance of each environment variable. The Jackknife test results and the variable contribution in Table 1 together can provide information for the relative importance of each environment variable. Second, we calculate the response curve of the MaxEnt model to the variation in each environment variable during 2000–2009. The response curve, together with the time series of the domain average environment variable, can provide more details of how a certain environment variable contributes to the increase/decrease of the predicted TC genesis probability.

#### Jackknife test

The Jackknife method was first introduced by Quenouille^72^ and named by Tukey.^73^ More details of the calculation process of the Jackknife test can be found in the supplement material of our previous work.^23^ The Jackknife test can provide the following information: if an environment variable has the largest gain when used in isolation, this variable has the most useful information compared with the rest of the variables; if an environment variable decreases, the MaxEnt model gains the most when it is excluded, this variable has the most information that the remaining variables do not have.

The Figures S8–S10 show the Jackknife test results of EP_WP_NI, SI_SP and NA, respectively. For EP_WP_NI, eta-850 has the largest gain when used in isolation, indicating that eta-850 has the most useful information among all the ten variables. The PI decreases the EP_WP_NI model gain when it is excluded, suggesting that PI has the most unique information among the ten variables. For SI_SP, eta-200 has the largest gain when used in isolation, meaning that eta-200 has the most useful information. The PI decreases the SI_SP model gain when it is excluded, suggesting that PI has the most information other variables do not have. For NA, eta-850 has the largest gain when used in isolation, providing the most useful information to the model. The PI decreases the NA model gain when it is excluded, providing the most unique information to the model. Note that eta-150, eta-200, eta-850 and eta-925 have comparable gain values when used in isolation for the three MaxEnt models, suggesting that the upper- and lower-level absolute vorticity are important to the three MaxEnt models. The results of the Jackknife test and the variable contribution in Table 1 together show that the PI and the upper and lower level absolute vorticity are essential to the MaxEnt model. Different from the above results, in our previous work,^23^ the PI has the most useful information and the most unique information in predicting global-scale TC genesis probability. Thus, the results in this study suggest that the upper and lower level absolute vorticity becomes more important to the MaxEnt model when predicting basin-wide TC genesis probability.

Previous studies have investigated the relationship between upper and lower-level vorticity and the TC genesis. Wu et al.^8^ pointed out that the tropical upper tropospheric trough (TUTT) in the North Pacific Ocean can affect TC genesis. Wang and Wu^9^ further revealed that TUTT can be identified with a vorticity maximum over the upper level of the atmosphere. Hsieh et al.^49^ revealed that the change in TC seed frequency over the Northwestern Pacific Ocean relies on the projection of changes in large-scale atmospheric convection on background absolute vorticity on both regional and global scales. These results suggest that the upper-level vorticity could affect the basin-wide TC genesis. Tippett et al.^20^ used several variables to construct a Poisson regression index for TC genesis, and they further showed that this index responds to low-level absolute vorticity to saturate when low-level absolute vorticity exceeds a threshold, which is essential for the good performance of this index. Hsieh et al.^46^ revealed that the convection process is not a dominant factor for Northwestern Pacific Ocean TC genesis under a high, low-level, low-frequency vorticity background but is essential for TC genesis under a low low-level, low-frequency vorticity background. Hsieh et al.^47^ further pointed out that the high-frequency part of low-level vorticity mainly affects the timing and location of Northwestern Pacific Ocean TC genesis. Ikehata and Satoh^48^ pointed out that northern hemisphere TC seed frequency is correlated with monthly mean lower-level vorticity and vertical velocity. These studies highlight the importance of low-level vorticity to TC genesis.

#### Response curve

The response curves of the three MaxEnt models are calculated following Qian et al.^23^ and presented in Figure S11. The physical meaning of the response curves and a summary of related previous studies are discussed in Qian et al.^23^ Unlike to our previous work, low-level absolute vorticity becomes important to MaxEnt models in this study. The response curve reveals that the TC genesis probability is asymmetric to the absolute zero vorticity. For example, given the same absolute vorticity level, e.g., 0.5 × 10-4 s^−1^ at 850 hPa, the predicted TC genesis probability in SI_SP is less than 0.2, while the predicted probability in NA and EP_WP_NI is more than 0.6. Such phenomenon suggests that the models regard that it is easier to generate TCs in the northern hemisphere than in the southern hemisphere to give the same absolute vorticity. For the northern hemisphere, the response curve of NA and EP_WP_NI implies that with the change of absolute vorticity, the predicted TC genesis may differ. For example, when absolute vorticity at 850 hPa changes from 0.4 × 10^−4^ s^−1^ to 0.5 × 10^−4^ s^−1^, the predicted probability in EP_EP_NI is decreased, while that is increased in NA.

In this study, we further determine the effect of each environment variable on the TC genesis probability under the benefit of the response curves. The Figure S12A presents the time series of the area-weighted average of eta-150 of each CMIP6 climate model under different SSP scenarios over the EP basin. The eta-150 shows a decreasing trend in all CMIP6 climate models under all SSP scenarios, and the value of eta-150 ranges from 0.485 × 10^−4^ s^−1^ to 0.455 × 10^−4^ s^−1^ (Figure S12A). The response curve of EP_WP_NI to eta-150 shows that when eta-150 falls between 0.25 × 10^−4^ s^−1^ to 1.0 × 10^−4^ s^−1^, decreasing eta-150 would increase the MaxEnt model predicted TC genesis probability. Therefore, compare Figures S11A and S12A, eta-150 has an overall increased effect on the predicted TC genesis probability because eta-150 will decrease in the future. The Figure S12B presents the time series of the area-weighted average of PI of each CMIP6 climate model under different SSP scenarios over the WP basin. The response curve of EP_WP_NI to PI shows that when PI is larger than 46, increasing PI would not increase the predicted TC genesis probability. Therefore, although the projected PI has an increasing trend, it would not increase the predicted TC genesis probability because PI is always larger than 46.

Table S3 summarizes the effect of each environment variable on the predicted TC genesis probability over each basin. Overall, although PI tends to be one of the most important environmental variables in the MaxEnt model, it only affects the predicted TC genesis over EP and SP. Some environment variables even have a different effect on the TC genesis probability, but the MaxEnt model seems insensitive to these variables. In other words, the MaxEnt model considers as many useful environmental variables as possible and predicts TC genesis in the future under the overall effect of all these environmental variables.

#### Multivariate environmental similarity surface (MESS)

MESS analysis is widely used in ecological studies to analyze the similarity between two environmental datasets.^52^ In the current work, we use it to investigate the similarity of the CMIP6 model-simulated TC genesis environment between two time periods.

We assume the environment variable dataset at a reference time period (in this study, this period is considered to be the historical runs of the CMIP6 models in the 1860s) is R, and $R_{i}$ is one environmental variable in R. The minimum value of $R_{i}$ is denoted as $\min_{R_{i}}$, and the maximum value of $R_{i}$ is denoted as $\max_{R_{i}}$. The model output at a target time period (in this study, this period is considered to be the SSP scenario runs of the CMIP6 models in the 2100s) of $R_{i}$ at a grid point x is denoted as $T_{ix}$. Let $p_{i}$ be the percent of grid points at the reference time period whose value of $R_{i}$ is smaller than $T_{ix}$. For a grid point x, the similarity S of $R_{i}$ between the reference and target time period is calculated using the following formulas:

(Equation 4) $$S=\left\{ \begin{array}{cc} \frac{T_{ix}-\min_{R_{i}}}{\max_{R_{i}}-\min_{R_{i}}}*100 & if\;p_{i}=0\\ 2*p_{i} & if\;0<p_{i}\leq 50\\ 2*\left(100-p_{i}\right) & if\;50<p_{i}<100\\ \frac{\max_{R_{i}}-T_{ix}}{\max_{R_{i}}-\min_{R_{i}}}*100 & if\;p_{i}=100 \end{array}\right.$$

There is no difference between the two datasets if S is 100; when S is greater than zero, the larger the S value is, the smaller the climate difference is between the future and the current scenarios. When S is less than zero, it indicates that there is at least one environmental variable that is out of the range of the reference dataset. In other words, a grid cell with negative MESS values means that the simulated TC genesis environment significantly changes at the target time period compared with the reference time period under the influence of climate change. Figure S13 shows the ensemble mean of the MESS values of the five CMIP6 models in the 2100s under the six SSP future scenarios. For example, there are negative MESS values near 180° and 30°S in Figure S13F, which means that the simulated TC genesis environment apparently changed in the 2100s compared to the 1860s.

### Quantification and statistical analysis

The maximum and minimum analysis is conducted to create Figure 2. The bar in Figure 2 represents the data range, with the lower edge of the bar representing the minimum and the upper edge representing the maximum.
