# Supplementary material for: Basin dependencies of tropical cyclone genesis environment and possible future changes revealed by machine learning methods
Source: iScience. 2025 Jan 3;28(2):111714. doi: 10.1016/j.isci.2024.111714 (PMC11795079; doi:10.1016/j.isci.2024.111714)
Supplement: Document S1. Figures S1–S13 and Tables S1–S3 [file mmc1.pdf]

iScience, Volume *28*

Supplemental information

Basin dependencies of tropical cyclone genesis environment and possible future changes revealed by machine learning methods

QiFeng Qian, YeFeng Chen, XiaoJing Jia, Hao Ma, and Wei Dong

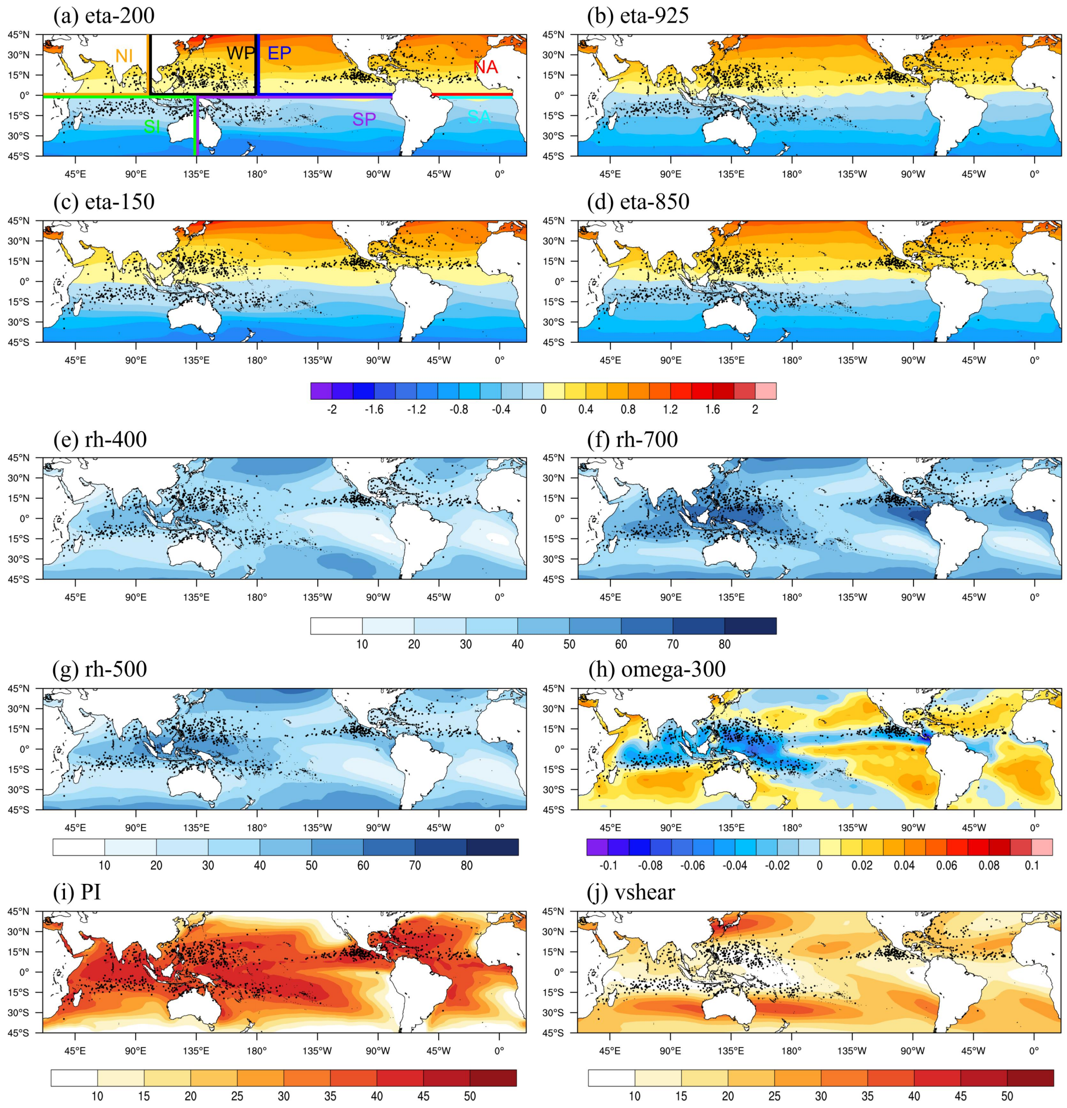

Figure S1 [Related to STAR Methods section] Environmental variables (color shading) and TC genesis locations (black dots) during the training period (2000-2009). The lines in (a) represent the definition of different ocean basins.

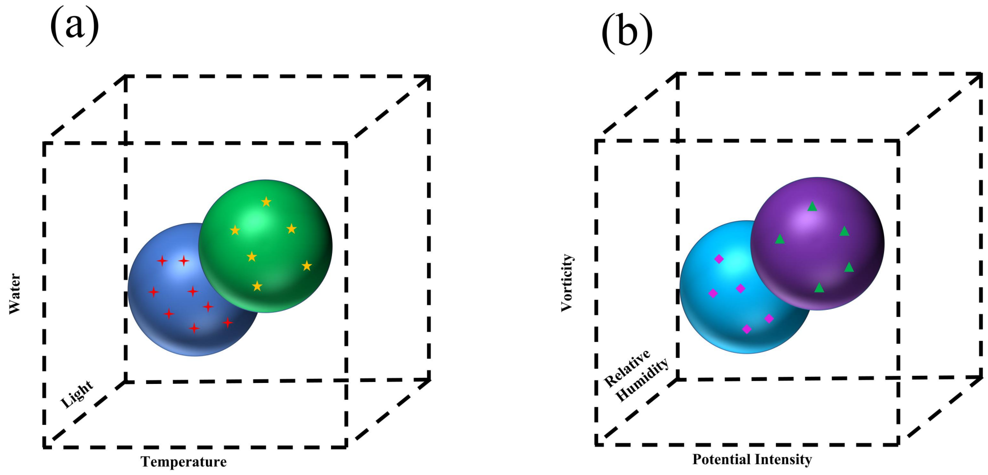

Figure S2 [Related to STAR Methods section] (a) Blue and green balls represent the envelope of environmental conditions within which a species can maintain a viable population. Red crosses and yellow stars denote individuals of a species. (b) Cyan and purple balls represent the envelope of environmental conditions of an ocean basin, within which TCs can be generated over this basin. The purple rhombus and green triangle represent the TCs that are generated in the basin.

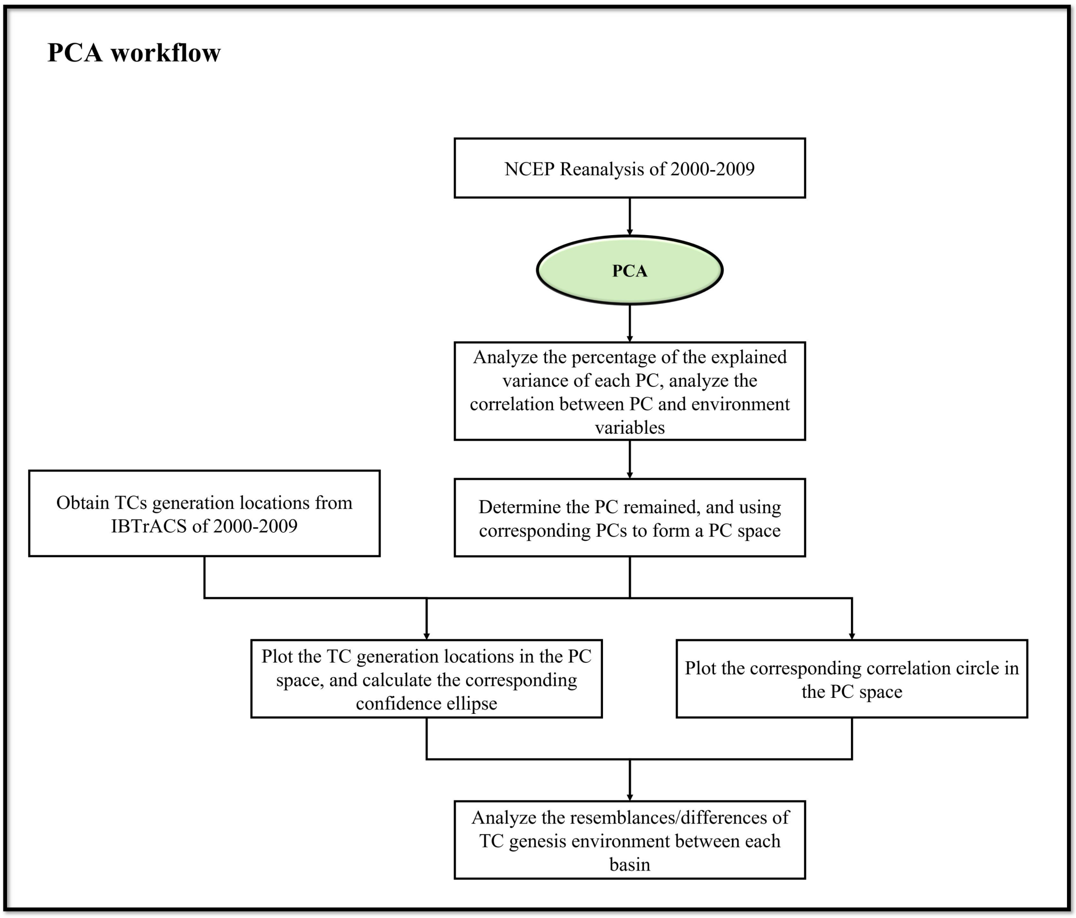

Figure S3 [Related to STAR Methods section] Diagram of the analysis workflow.

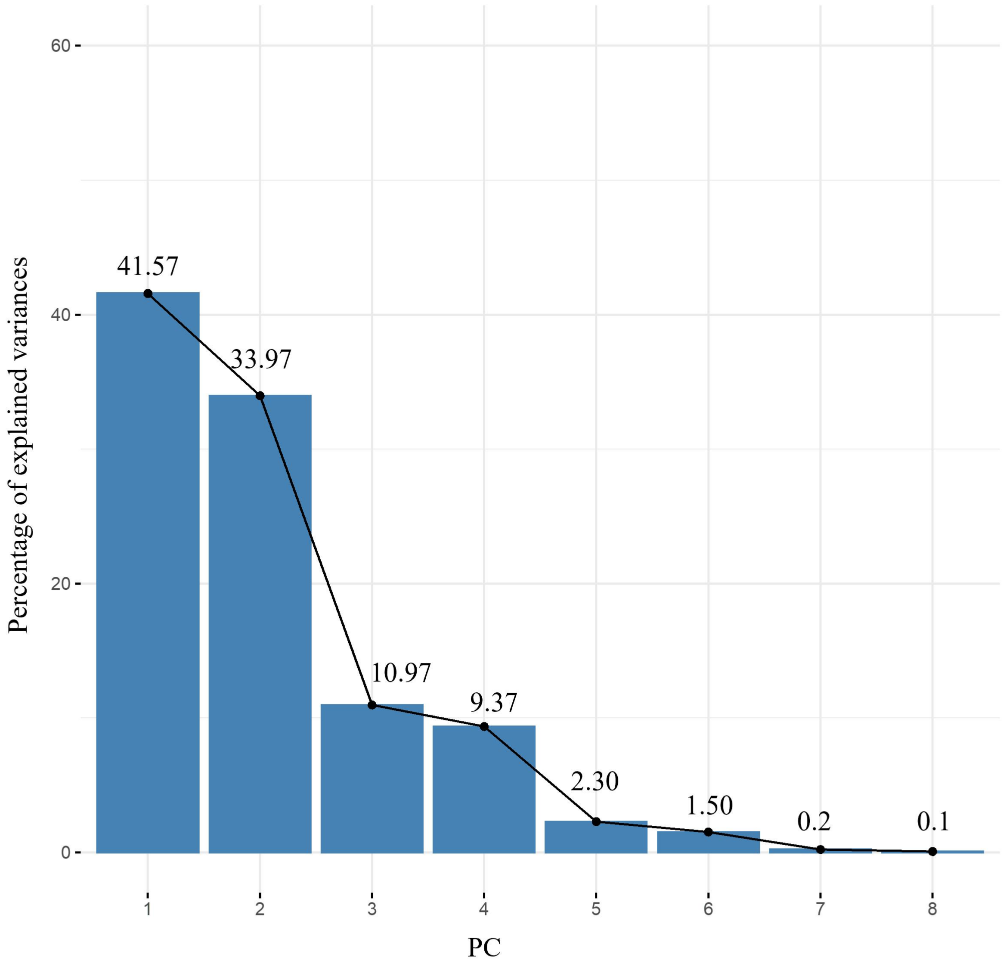

Figure S4 [Related to STAR Methods section] The y-axis represents the percentage of the explained variance, and the x-axis represents the PCs. The numbers above each bar denote the explained variances of each PC.

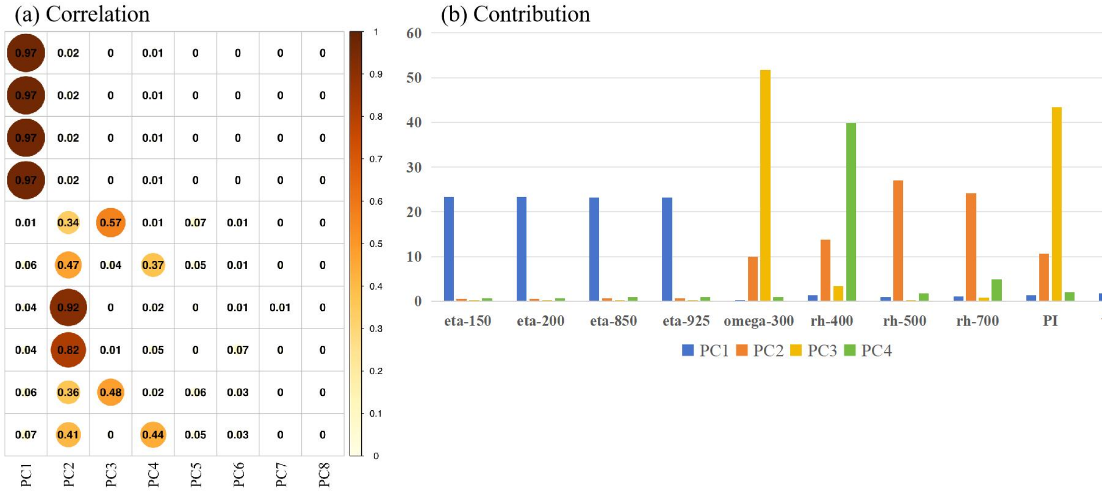

Figure S5 [Related to STAR Methods section] (a) Correlation coefficient heatmap of the PCs and the original environmental variables. (b) Contribution of each environmental variable to PCs (%).

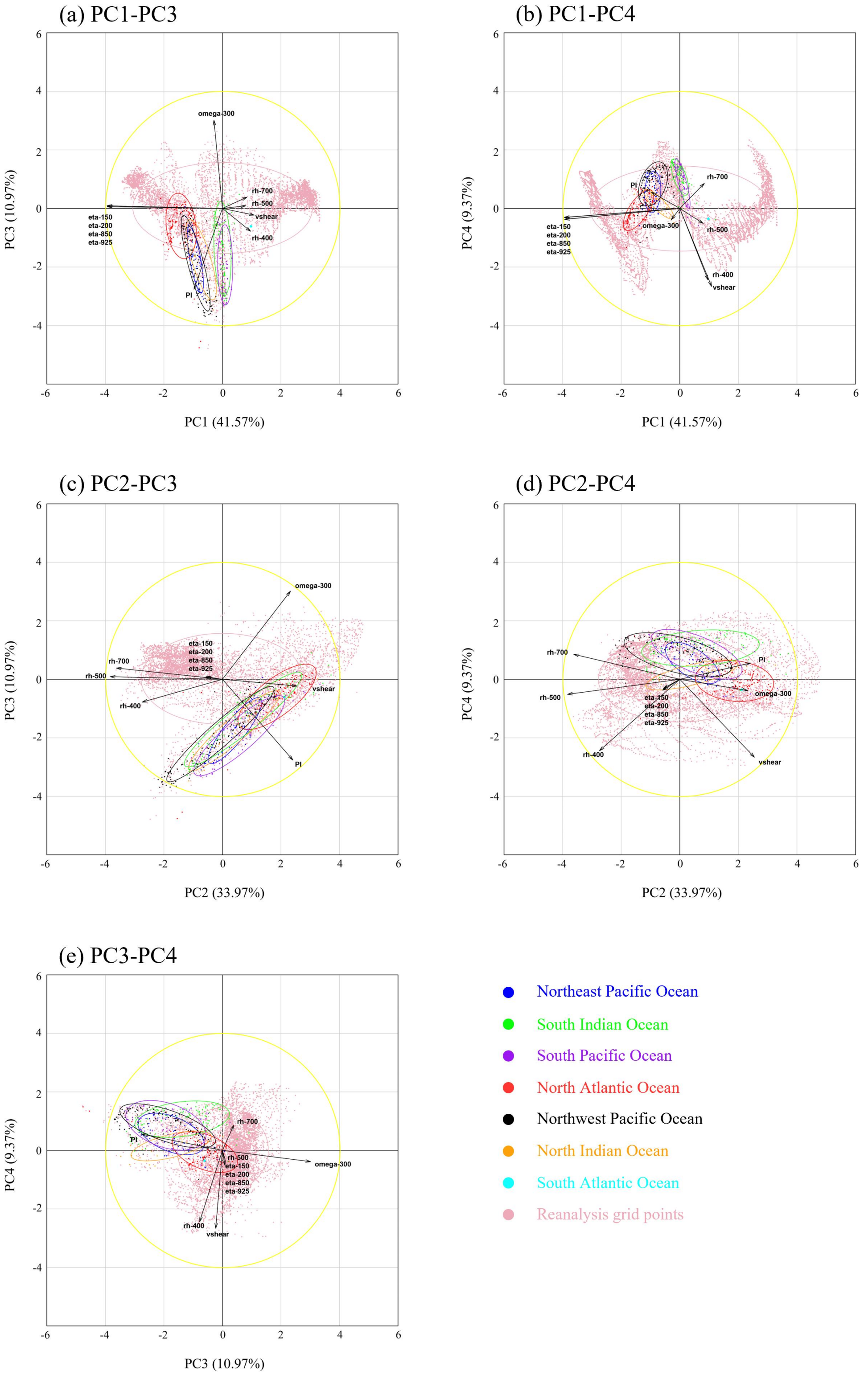

Figure S6 [Related to STAR Methods section] TC genesis locations of different ocean basins and reanalysis grid points (dots) and corresponding confidence ellipses (95% confidence level) during 2000-2009 in different PC spaces formed by PC1, PC2, PC3 and PC4. The yellow circle and black vector present the results from the correlation circle, which is enlarged by a factor of four for better presentation.

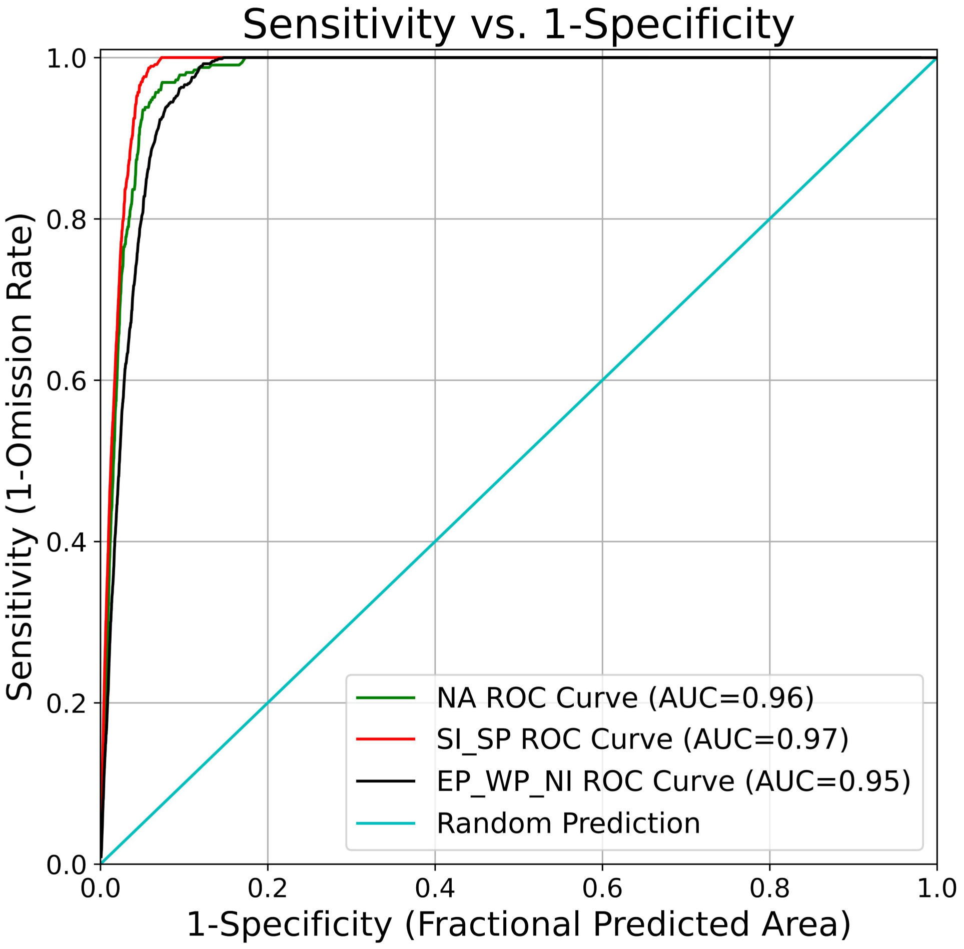

Figure S7 [Related to main text and STAR Methods section] The ROC curve and the area under the ROC curve (AUC) of the three MaxEnt model.

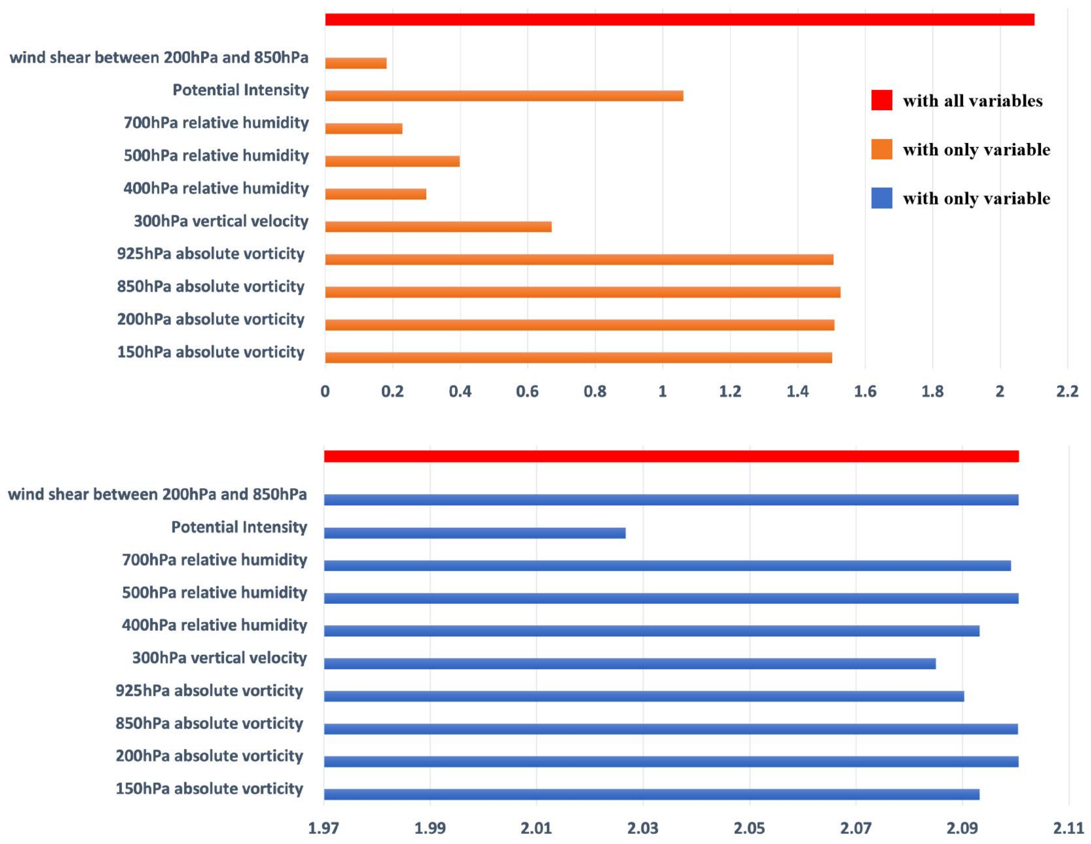

Figure S8 [Related to STAR Methods section] The results of the Jackknife test of the EP_WP_NI. The red bar represents the gain using all variables. The orange bars denote the gain using only a certain variable. The blue bars represent the gain without a certain variable. The abscissa is the gain.

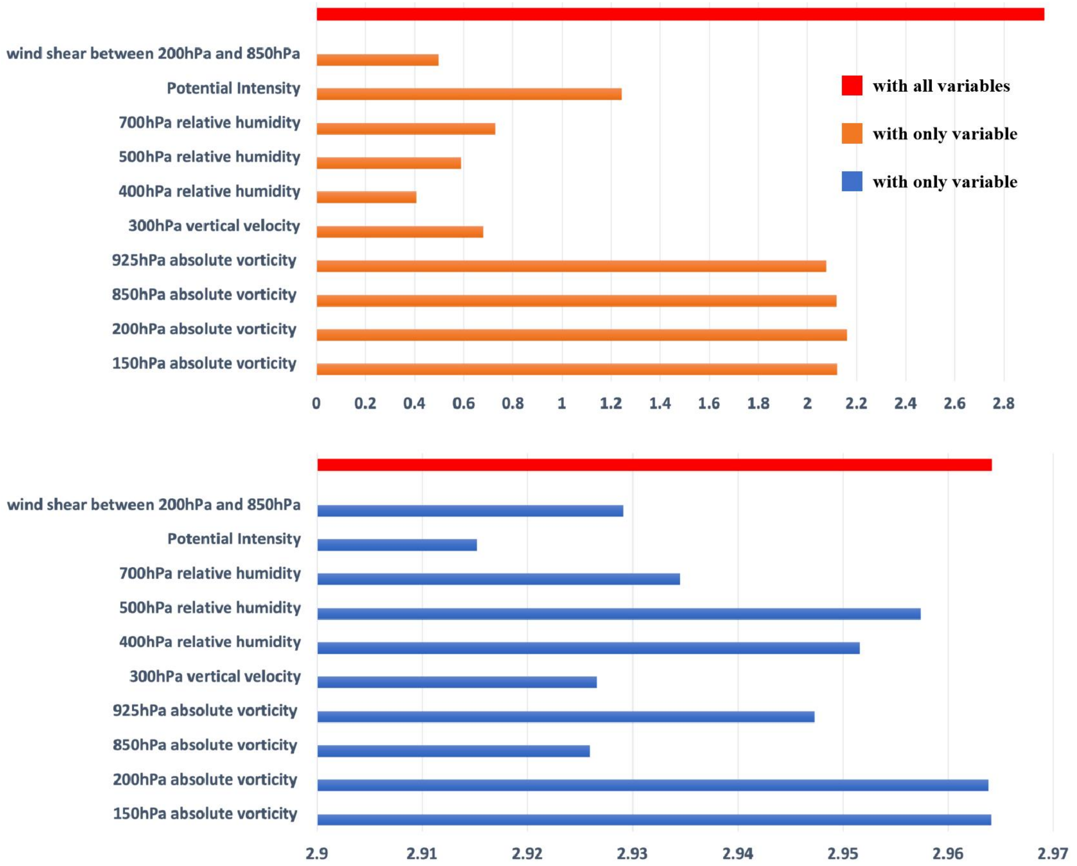

Figure S9 [Related to STAR Methods section] The results of the Jackknife test of the SI_SP. The red bar represents the gain using all variables. The orange bars denote the gain using only a certain variable. The blue bars represent the gain without a certain variable. The abscissa is the gain.

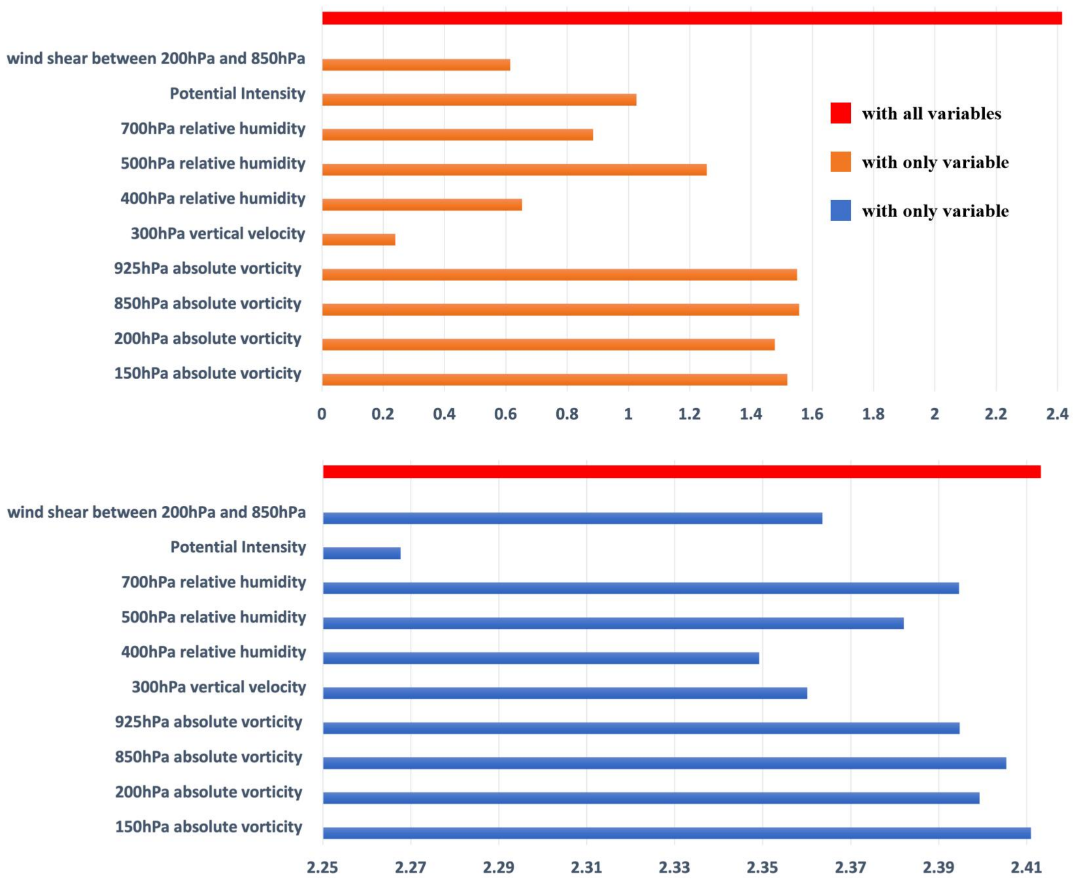

Figure S10 [Related to STAR Methods section] The results of the Jackknife test of the NA. The red bar represents the gain using all variables. The orange bars denote the gain using only a certain variable. The blue bars represent the gain without a certain variable. The abscissa is the gain.

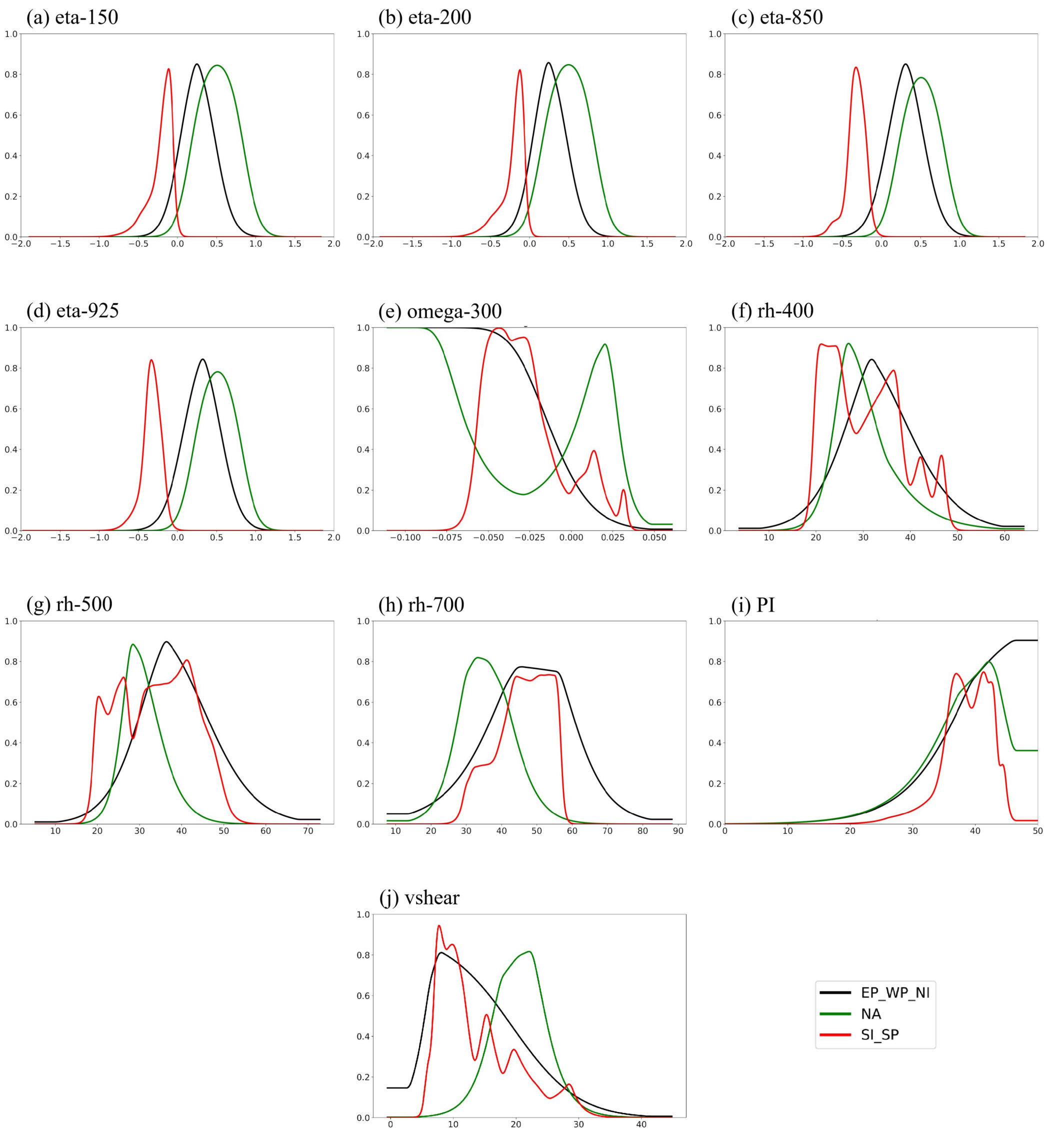

Figure S11 [Related to STAR Methods section] Response curve. The response curves of (a) 150 hPa absolute vorticity (unit: 1×10^{-4} s^{-1}), (b) 200 hPa absolute vorticity (unit: 1×10^{-4} s^{-1}), (c) 850 hPa absolute vorticity (unit: 1×10^{-4} s^{-1}), (d) 925 hPa absolute vorticity (unit: 1×10^{-4} s^{-1}), (e) 300 hPa vertical velocity (unit: Pa/s), (f) 400 hPa relative humidity (unit: %), (g) 500 hPa relative humidity (unit: %), (h) 700 hPa relative humidity (unit: %), (i) PI (unit: m s^{-1}) and (j) vertical wind shear between 200 hPa and 500 hPa. The abscissa is the value varying from its minimum to its maximum for the training dataset, and the ordinate is the TC genesis probability.

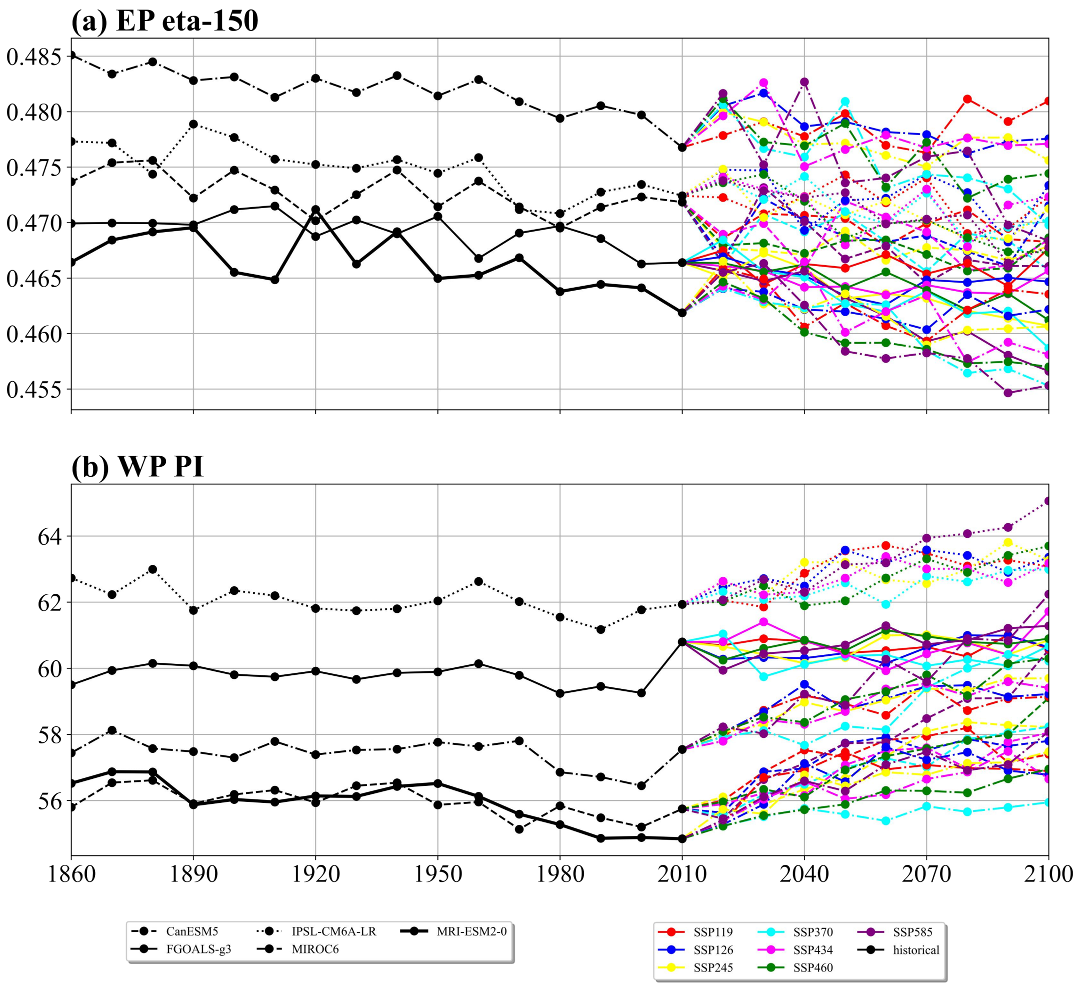

Figure S12 [Related to STAR Methods section] The area-weighted average (a) eta-150 (unit: 1×10^{-4} s^{-1}) and (b) PI (unit: m s^{-1}) of each CMIP6 climate model under each SSP scenario over the EP basin.

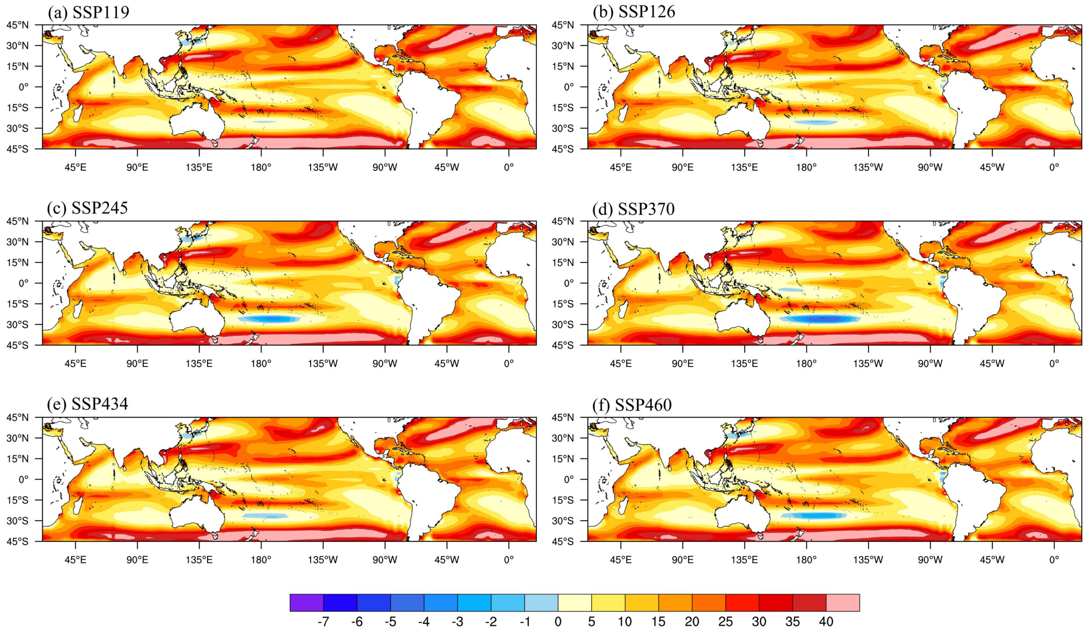

Figure S13 [Related to main text and STAR Methods section] The ensemble mean of the MESS values of the CMIP6 models in the 2100s under different SSP future scenarios. The SSP 585 scenario is provided in the main text.

Table S1 [Related to STAR Methods section] List of CMIP6 models used in this study.

Model	Institute	Nation	Atmospheric resolution	Reference
CanESM5	Canadian Centre for Climate Modeling and Analysis	Canada	2.8°×2.8°	Swart et al. (2019)
FGOALS-g3	The Institute of Atmospheric Physics	China	2°×2°	Li et al. (2020)
IPSL-CM6A-LR	Institut Pierre Simon Laplace	France	1.25°×2.5°	Hourdin et al. (2020)
MIROC6	Center for Climate System Research<br>University of Tokyo<br>Japan Agency for Marine-Earth Science and Technology<br>National Institute for Environmental Studies	Japan	1.4°×1.4°	Tatebe et al. (2019)
MRI-ESM2-0	The Meteorological Research Institute	Japan	T159 (~120 km)	Yukimoto et al. (2019)

Table S2 [Related to main text and STAR Methods section] The spatial correlation coefficients between the TC genesis probability predicted by the MaxEnt model (denoted as MaxEnt), the EM GPI, the MK GPI and the observations for the periods of 1990 – 1999, 2000-2009 and 2010 – 2019. The spatial correlation coefficients between the TC genesis probability predicted by the transferred MaxEnt model for the period of 2000-2009 (denoted as CMIP ENS).

	NA	EP	WP	NI	SP	SI
1990-1999 MaxEnt	0.62	0.76	0.69	0.55	0.76	0.76
1990-1999 EM GPI	0.31	0.56	0.54	0.30	0.72	0.74
1990-1999 MK GPI	0.27	0.77	0.65	0.33	0.75	0.75
2000-2009 MaxEnt	0.65	0.73	0.72	0.55	0.81	0.81
2000-2009 EM GPI	0.30	0.66	0.69	0.34	0.69	0.77
2000-2009 MK GPI	0.20	0.84	0.76	0.28	0.70	0.73
2010-2019 MaxEnt	0.60	0.81	0.72	0.50	0.70	0.71
2010-2019 EM GPI	0.30	0.55	0.65	0.36	0.62	0.70
2010-2019 MK GPI	0.21	0.80	0.75	0.33	0.63	0.62
2000-2009 CMIP ENS	0.59	0.70	0.67	0.56	0.74	0.77

Table S3 [Related to STAR Methods section] The effect of each environment variable on the predicted TC genesis probability. The mean time series (averaged across all CMIP6 models) of a certain environment variable over a certain basin under a certain SSP scenario is calculated first. Then, the significance of its linear trend ($p<0.05$) is tested. "/" denotes the linear trend is not significant. Finally, the mean time series is compared with the corresponding response curve to obtain the TC genesis probability increase/decrease value. Positive numbers are marked in red and negative numbers are marked in blue. The zero number means that although a specific environment variable has a linear trend, such a trend has a limited effect on the TC genesis probability.

		eta-150	eta-200	eta-850	eta-925	omega-300	rh-400	rh-500	rh-700	vpot	vshear
NA	ssp119	0.00	0.00	0.00	-0.10	-0.01	0.01	0.00	0.02	0.00	0.02
	ssp126	0.00	0.00	0.00	-0.10	-0.01	0.02	0.01	0.03	0.00	0.02
	ssp245	0.00	0.00	0.00	-0.10	-0.02	0.04	0.02	0.05	0.00	0.03
	ssp370	0.01	0.00	0.00	-0.10	-0.04	0.07	0.04	0.08	0.00	0.04
	ssp434	0.00	0.00	0.00	-0.10	-0.02	0.04	0.02	0.04	0.00	0.02
	ssp460	0.00	0.00	0.00	-0.10	-0.03	0.05	0.03	0.07	0.00	0.03
	ssp585	0.02	0.01	-0.04	-0.10	-0.05	0.09	0.05	0.10	0.00	0.04
EP	ssp119	0.01	0.01	0.00	0.00	0.01	-0.01	-0.03	-0.08	0.00	-0.01
	ssp126	0.01	0.01	0.00	0.00	0.01	-0.01	-0.04	-0.08	0.00	-0.01
	ssp245	0.01	0.01	0.00	0.00	0.02	-0.02	-0.07	0.00	0.00	-0.02
	ssp370	0.02	0.01	-0.01	0.00	0.04	-0.05	-0.12	0.00	0.02	-0.03
	ssp434	0.01	0.01	0.00	0.00	0.02	-0.03	-0.06	0.00	0.00	-0.01
	ssp460	0.02	0.01	0.00	0.00	0.02	-0.02	-0.08	0.00	0.00	-0.02
	ssp585	0.03	0.02	-0.01	0.00	0.05	-0.07	-0.15	0.00	0.02	-0.02
WP	ssp119	0.01	0.01	-0.08	0.00	-0.01	-0.03	-0.02	0.01	0.00	-0.01
	ssp126	0.01	0.01	-0.08	0.00	-0.01	-0.03	-0.02	0.02	0.00	-0.01
	ssp245	0.01	0.01	0.00	0.00	-0.02	-0.03	-0.04	0.02	/	-0.02
	ssp370	0.02	0.03	0.00	0.00	-0.04	-0.02	-0.05	0.02	0.00	-0.02
	ssp434	0.01	0.01	-0.08	0.00	-0.02	-0.02	-0.03	0.02	0.00	-0.01
	ssp460	0.01	0.01	0.00	0.00	-0.02	-0.02	-0.05	0.01	0.00	-0.02

	**ssp585**	0.02	0.03	0.00	0.00	-0.02	-0.02	-0.08	0.00	0.00	-0.02
	**ssp119**	0.00	0.00	0.00	0.00	0.01	-0.04	0.00	0.00	0.00	0.00
	**ssp126**	0.00	0.00	0.00	0.00	0.01	-0.04	-0.01	0.00	/	0.00
	**ssp245**	0.01	0.07	0.00	0.00	0.02	-0.04	-0.01	0.00	0.00	0.00
**NI**	**ssp370**	0.01	0.07	0.00	0.00	0.03	-0.04	-0.02	0.00	/	0.00
	**ssp434**	0.01	0.07	0.00	0.00	0.02	-0.04	-0.02	0.00	0.00	0.00
	**ssp460**	0.01	0.07	0.00	0.00	0.02	-0.04	-0.02	0.00	0.00	-0.01
	**ssp585**	0.01	0.07	-0.01	-0.01	0.04	-0.04	-0.03	0.00	0.00	-0.01
	**ssp119**	0.02	0.01	0.00	0.00	0.01	-0.04	0.00	-0.01	0.00	0.00
	**ssp126**	0.02	0.01	0.00	0.00	0.01	-0.05	0.00	-0.01	0.00	0.00
	**ssp245**	0.04	0.02	0.00	0.00	0.01	-0.07	0.00	-0.02	0.00	-0.01
**SI**	**ssp370**	0.05	0.02	0.00	0.00	0.02	-0.08	0.00	-0.02	0.00	-0.02
	**ssp434**	0.03	0.02	0.00	0.00	0.01	-0.06	0.00	-0.01	0.00	0.00
	**ssp460**	0.05	0.02	0.00	0.00	0.02	-0.07	0.00	-0.02	0.00	-0.01
	**ssp585**	0.07	0.03	0.00	0.00	0.03	-0.11	0.00	-0.03	0.00	-0.02
	**ssp119**	0.02	0.01	0.00	0.00	-0.01	-0.01	0.00	-0.02	0.00	-0.04
	**ssp126**	0.02	0.01	0.00	0.00	-0.01	-0.01	0.00	0.00	0.00	-0.05
	**ssp245**	0.03	0.01	0.00	0.00	-0.02	0.03	0.01	0.00	0.00	-0.07
**SP**	**ssp370**	0.05	0.02	-0.01	0.00	-0.04	0.07	0.02	-0.01	-0.02	-0.08
	**ssp434**	0.02	0.01	0.00	0.00	-0.01	0.03	0.01	0.00	-0.02	-0.06
	**ssp460**	0.04	0.01	0.00	0.00	-0.03	0.04	0.01	-0.01	-0.02	-0.07
	**ssp585**	0.06	0.02	-0.01	0.00	-0.04	0.07	0.03	-0.01	-0.02	-0.10

Supplement Information References

Swart, N. C., Cole, J. N., Kharin, V. V., Lazare, M., Scinocca, J. F., Gillett, N. P., ... & Winter, B. (2019). The Canadian earth system model version 5 (CanESM5. 0.3). Geoscientific Model Development, 12(11), 4823-4873.

Li, L., Yu, Y., Tang, Y., Lin, P., Xie, J., Song, M., ... & Wei, J. (2020). The flexible global ocean-atmosphere-land system model grid-point version 3 (FGOALS-g3): description and evaluation. Journal of Advances in Modeling Earth Systems, 12(9), e2019MS002012.

Hourdin, F., Rio, C., Grandpeix, J. Y., Madeleine, J. B., Cheruy, F., Rochetin, N., ... & Ghattas, J. (2020). LMDZ6A: The atmospheric component of the IPSL climate model with improved and better tuned physics. Journal of Advances in Modeling Earth Systems, 12(7), e2019MS001892.

Tatebe, H., Ogura, T., Nitta, T., Komuro, Y., Ogochi, K., Takemura, T., ... & Kimoto, M. (2019). Description and basic evaluation of simulated mean state, internal variability, and climate sensitivity in MIROC6. Geoscientific Model Development, 12(7), 2727-2765.

Yukimoto, S., Kawai, H., Koshiro, T., Oshima, N., Yoshida, K., Urakawa, S., ... & Ishii, M. (2019). The Meteorological Research Institute Earth System Model version 2.0, MRI-ESM2. 0: Description and basic evaluation of the physical component. Journal of the Meteorological Society of Japan. Ser. II.
